# Supplementary material for: Polo kinase recruitment via the constitutive centromere-associated network at the kinetochore elevates centromeric RNA
Source: PLoS Genet. 2020 Aug 18;16(8):e1008990. doi: 10.1371/journal.pgen.1008990 (PMC7455000; doi:10.1371/journal.pgen.1008990)
Supplement: S1 Table — (PDF) [file pgen.1008990.s001.pdf]

Table S1. Strains and plasmids used in this study.

Saccharomyces cerevisiae strains:

Strain	Relevant genotype	Reference
BY4741	*MATa his3Δ1 leu2Δ0 met15Δ0 ura3Δ0*	Brachmann et al. 1998
W8164-2B	*MATα CEN1-16::Gal-Kl-URA3*	Reid et al. 2011
GFP strains	*MATa his3Δ1 leu2Δ0 met15Δ0 ura3Δ0 ORF- GFP::HIS3MX6*	Huh et al. 2003
Deletion strains	*MATα his3Δ1 leu2Δ0 lys2Δ0 ura3Δ0 orfΔ::KANMX*	Winzeler et al. 1999
Deletion strains	*MATa his3Δ1 leu2Δ0 met15Δ0 ura3Δ0 orfΔ::KANMX*	Winzeler et al. 1999
YMB10337	*MATα ura3-52 lys2-801 ade2-101 trp1Δ63 his3Δ200 leu2Δ1 CFIII (CEN3L.YPH278) HIS3 SUP11 cse4-9SA-3HA::URA3*	Mishra et al. 2019
T548	*MATa his3Δ1 leu2Δ0 met15Δ0 ura3Δ0 CSE4-GFP (internal)::HIS3MX6*	Mishra et al. 2019
T664	*MATα his3Δ1 leu2Δ0 lys2Δ0 ura3Δ0 CSE4-YFP (internal)::HIS3MX6*	Mishra et al. 2019
PT63-12B	*MATa ADE2 leu2-3,112 his3-11,15 URA3 LYS2 can1-100 trp1-1 cdc20::prMET3-CDC20::TRP1 MTW1-YFP*	This study
PT257	*MATa ADE2 leu2-3,112 his3-11,15 URA3 LYS2 can1-100 trp1-1 cdc20::prMET3-CDC20::TRP1 MTW1-YFP SPC110-CFP::KANMX*	This study
T16	*MATα ADE2 leu2-3,112 TRP1 lys2Δ MTW1-YFP RAD5 mad1Δ::KANMX*	Olafsson & Thorpe 2016
T17	*MATα ADE2 leu2-3,112 TRP1 lys2Δ MTW1-YFP RAD5 mad3Δ::KANMX*	This Study
T18	*MATα ADE2 leu2-3,112 TRP1 lys2Δ MTW1-YFP RAD5 bub1Δ::KANMX*	This Study
T19	*MATα ADE2 leu2-3,112 TRP1 lys2Δ MTW1-YFP RAD5 bub3Δ::KANMX*	This Study
T414	*MATa NUF2-GFP::HIS3 mad3Δ::KANMX*	Olafsson & Thorpe 2015
T415	*MATa NNF1-GFP::HIS3 mad3Δ::KANMX*	Olafsson & Thorpe 2015
T416	*MATa MTW1-GFP::HIS3 mad3Δ::KANMX*	Olafsson & Thorpe 2015
T417	*MATa CDC14-GFP::HIS3 mad3Δ::KANMX*	Olafsson & Thorpe 2015
T418	*MATa KRE28-GFP::HIS3 mad3Δ::KANMX*	Olafsson & Thorpe 2015
T419	*MATa NSL1-GFP::HIS3 mad3Δ::KANMX*	Olafsson & Thorpe 2015
T420	*MATa CEP3-GFP::HIS3 mad3Δ::KANMX*	Olafsson & Thorpe 2015
T421	*MATa DAD3-GFP::HIS3 mad3Δ::KANMX*	Olafsson & Thorpe 2015
T422	*MATa BIK1-GFP::HIS3 mad3Δ::KANMX*	Olafsson & Thorpe 2015
T423	*MATa BUB3-GFP::HIS3 mad3Δ::KANMX*	Olafsson & Thorpe 2015
T424	*MATa MIF2-GFP::HIS3 mad3Δ::KANMX*	Olafsson & Thorpe 2015
T425	*MATa CBF1-GFP::HIS3 mad3Δ::KANMX*	Olafsson & Thorpe 2015
T426	*MATa SLI15-GFP::HIS3 mad3Δ::KANMX*	Olafsson & Thorpe 2015
T427	*MATa AME1-GFP::HIS3 mad3Δ::KANMX*	Olafsson & Thorpe 2015
T428	*MATa MCM21-GFP::HIS3 mad3Δ::KANMX*	Olafsson & Thorpe 2015
T429	*MATa CTF19-GFP::HIS3 mad3Δ::KANMX*	Olafsson & Thorpe 2015
T430	*MATa STU2-GFP::HIS3 mad3Δ::KANMX*	Olafsson & Thorpe 2015
T431	*MATa DSN1-GFP::HIS3 mad3Δ::KANMX*	Olafsson & Thorpe 2015
T594	*MATa BUB1-GFP::HIS3 mad3Δ::KANMX*	This Study
T595	*MATa SPC105-GFP::HIS3 mad3Δ::KANMX*	This Study
T596	*MATa NDC80-GFP::HIS3 mad3Δ::KANMX*	This Study
T603	*MATa DAD1-GFP::HIS3 mad3Δ::KANMX*	This Study
T604	*MATa DAD4-GFP::HIS3 mad3Δ::KANMX*	This Study
T605	*MATa BIM1-GFP::HIS3 mad3Δ::KANMX*	This Study
T607	*MATα ADE2 leu2-3,112 his3-11,15 URA3 LYS2 can1-100 trp1-1 cdc20::prMET3-CDC20::TRP1 MTW1-YFP Turq2-TUB1::HIS3MX6*	This Study
T621	*MATa ADE2 trp1-1 LYS2 his3-11,15 ura3-1 SPC42-RFP::HYGMX DAD4-YFP::NATMX Turq2-TUB1::HIS3MX6*	This Study
YMB54	*MATa ura3-52 lys2-801 ade2-101 his3D200 leu2D1 spt4-138*	Basrai et al. 1996

PT285-7B	*MATα his3Δ1 leu2Δ0 trp1-1 cdc20::prMET3-CDC20::TRP1 SMC3-CFP::KANMX CSE4-YFP (internal)::HIS3MX6*	This study
T682	*MATα CEN1-16::Gal-Kl-URA3 spt4Δ::KANMX*	This study
T360	*MAT***a** *CEN1-16::Gal-Kl-URA3 MTW1-YFP::HIS3*	This study
T738	*MAT***a** *CEN1-16::Gal-Kl-URA3 MTW1-YFP::HIS3 spt4Δ::KANMX*	This study
T739	*MAT***a** *ADE2 leu2-3,112 his3-11,15 URA3 LYS2 can1-100 trp1-1 cdc20::prMET3-CDC20::TRP1 MTW1-YFP spt4::KANMX*	This study
T692	*MAT***a** *ADE2 trp1-1 LYS2 his3-11,15 SPC42-RFP::HYGMX DAD4-YFP::NATMX Turq2-TUB1::URA3*	This study
T670	*MATα his3Δ1 leu2Δ0 lys2Δ0 ura3Δ0 CSE4-YFP (internal)::HIS3MX6 spt4Δ::KANMX*	This study
T671	*MAT***a** *his3Δ1 leu2Δ0 met15Δ0 ura3Δ0 AME1-GFP::HIS3MX6 spt4Δ::KANMX*	This study
T672	*MAT***a** *his3Δ1 leu2Δ0 met15Δ0 ura3Δ0 MTW1-GFP::HIS3MX6 spt4Δ::KANMX*	This study
T673	*MAT***a** *his3Δ1 leu2Δ0 met15Δ0 ura3Δ0 KRE28-GFP::HIS3MX6 spt4Δ::KANMX*	This study
T674	*MAT***a** *his3Δ1 leu2Δ0 met15Δ0 ura3Δ0 NDC80-GFP::HIS3MX6 spt4Δ::KANMX*	This study
T675	*MAT***a** *his3Δ1 leu2Δ0 met15Δ0 ura3Δ0 DAD3-GFP::HIS3MX6 spt4Δ::KANMX*	This study

List of plasmids:		
**Plasmid**	**Description (promoter, genetic construct, selection marker)**	**Reference**
pHT4	*pCUP1 GBP LEU2*	Olafsson & Thorpe 2015
pHT10	*pCUP1 MTW1-GBP LEU2*	Olafsson & Thorpe 2015
pHT99	*pCUP1 NAT*	Olafsson & Thorpe 2015
pHT103	*pGAL1 LEU2*	Olafsson & Thorpe 2016
pHT199	*pGAL1 GBP LEU2*	Olafsson & Thorpe 2016
pHT208	*pCUP1 DAD2 LEU2*	Berry et al. 2016
pHT234	*pCUP1 DAD2-GBP LEU2*	Berry et al. 2016
pHT210	*pCUP1 NUF2 LEU2*	Olafsson & Thorpe 2015
pHT211	*pCUP1 NUF2-GBP LEU2*	Olafsson & Thorpe 2015
pHT296	*pCUP1 MTW1 LEU2*	Olafsson & Thorpe 2015
pHT310	*pCUP1 CBF1 LEU2*	Olafsson & Thorpe 2015
pHT311	*pCUP1 CTF19 LEU2*	Olafsson & Thorpe 2015
pHT312	*pCUP1 CTF19-GBP LEU2*	Olafsson & Thorpe 2015
pHT313	*pCUP1 MIF2 LEU2*	Olafsson & Thorpe 2015
pHT314	*pCUP1 MIF2-GBP LEU2*	Olafsson & Thorpe 2015
pHT318	*pCUP1 CNN1 LEU2*	Olafsson & Thorpe 2015
pHT319	*pCUP1 CNN1-GBP LEU2*	Olafsson & Thorpe 2015
pHT320	*pCUP1 GBP-CNN1 LEU2*	This study
pHT335	*pCUP1 SKP1 LEU2*	Olafsson & Thorpe 2015
pHT336	*pCUP1 SKP1-GBP LEU2*	Olafsson & Thorpe 2015
pHT337	*pCUP1 CTF3 LEU2*	Olafsson & Thorpe 2015
pHT338	*pCUP1 CTF3-GBP LEU2*	Olafsson & Thorpe 2015
pHT339	*pCUP1 KRE28 LEU2*	Olafsson & Thorpe 2015
pHT340	*pCUP1 KRE28-GBP LEU2*	Olafsson & Thorpe 2015
pHT341	*pCUP1 CHL4 LEU2*	Olafsson & Thorpe 2015
pHT342	*pCUP1 CHL4-GBP LEU2*	Olafsson & Thorpe 2015
pHT343	*pCUP1 GBP-CBF1 LEU2*	This study
pHT425	*pCUP1 CDC5-GBP LEU2*	Mishra et al. 2019
pHT426	*pCUP1 CDC5 LEU2*	This study

pHT442	*pCUP1 cdc5-K110A-GBP LEU2 (cdc5-kd-GBP)*	Mishra et al. 2019
pHT444	*pCUP1 cdc5-T242A-GBP LEU2 (cdc5-kd-GBP)*	This study
pHT503	*pCUP1 cdc5-T242A-WHK/FAM-GBP LEU2*	This study
pHT504	*pCUP1 CDC5-WHK/FAM-GBP-RFP LEU2*	This study
pHT568	*pGAL1 CDC5ΔC-AME1 LEU2*	This study
pHT569	*pGAL1 cdc5ΔC-K110A-AME1 LEU2 (cdc5ΔC-kd-AME1)*	This study
pHT573	*pGAL1 CDC5 LEU2*	This study
pHT574	*pGAL1 cdc5-K110A LEU2*	This study
pHT580	*pGAL1 CDC5ΔC-GBP LEU2*	This study
pHT581	*pGAL1 cdc5ΔC-K110A-GBP LEU2 (cdc5ΔC-kd-GBP)*	This study
pHT680	*pGAL1 PSH1 NAT*	This study
pHT632	*pGAL1 BIM1 NAT*	This study
pHT636	*pGAL1 BIK1 NAT*	This study
pHT781	*pGAL1 CDC5ΔC-AME1 HYG*	This study
pHT782	*pGAL1 cdc5ΔC-K110A-AME1 HYG (cdc5ΔC-kd-AME1)*	This study
pHT775	*pGAL1 CBF1 NAT*	This study
pHT723	*pCUP1 SPT4 NAT*	This study
pHT724	*pGAL1 SPT4 NAT*	This study
pHT684	*pGAL1 ame1-7A LEU2*	This study
pHT686	*pGAL1 CDC5ΔC-ame1-7A LEU2*	This study
