# Supplementary material for: Polo kinase recruitment via the constitutive centromere-associated network at the kinetochore elevates centromeric RNA
Source: PLoS Genet. 2020 Aug 18;16(8):e1008990. doi: 10.1371/journal.pgen.1008990 (PMC7455000; doi:10.1371/journal.pgen.1008990)
Supplement: S2 Table — (PDF) [file pgen.1008990.s002.pdf]

Table S2. Primers used for reverse transcription PCR

Gene or CEN RNA	Primer name (Forward primer) 5' > 3'	Primer name (Reverse primer) 5' > 3'	Product size	Reference
CEN1	OWYY473<br>TGTAATGATTTAAGTCTTGTCACATGA	OWYY474<br>AAAATACTTTGACTGCTTCGGAA	243 bp	Ling and Yuen 2019
CEN3	OWYY1025<br>ACATGGCATGGCGATCAGCG	OWYY1027<br>TGAGCAAAACTTCCACCAGTAAACG	263 bp	Ling and Yuen 2019
CEN8	OWYY1044<br>AACTCCAACAATTACACATCCACAAAACG	OWYY1045<br>AGAGATTTGGGCTTGAGGAAGCG	297 bp	Ling and Yuen 2019
CDC28	CDC28-F<br>CTTGAGAAAGTCGGTGAAGG	CDC28-R<br>GATTCTTGGAAGTAGGGGTG	0.86 kb	Ohkuni and Kitagawa 2011
