# Supplementary figures and images for: Polo kinase recruitment via the constitutive centromere-associated network at the kinetochore elevates centromeric RNA
Source: PLoS Genet. 2020 Aug 18;16(8):e1008990. doi: 10.1371/journal.pgen.1008990 (PMC7455000; doi:10.1371/journal.pgen.1008990)

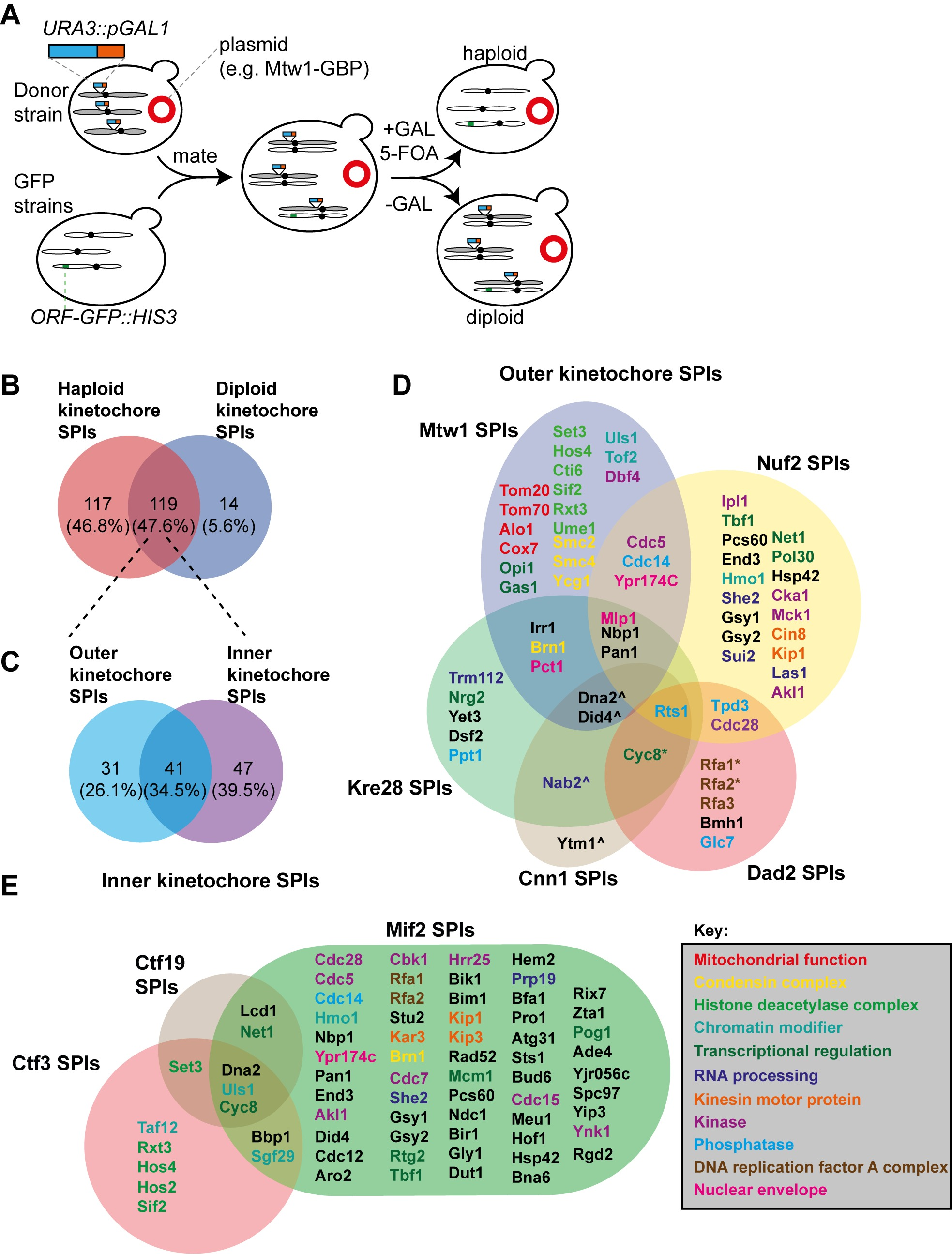

Supplement: S1 Fig — (A) A schematic of the SPI method. A plasmid containing a gene of interest fused to GBP (e.g. MTW1-GBP) or control plasmids are introduced into a universal donor strain (UDS), containing a URA3 locus and a conditional centromere on every chromosome. The UDS is mated with arrayed GFP strains, generating heterozygous diploid strains. For haploid SPI screens, the selective ploidy ablation technique is used to destabilize and select against the UDS chromosomes by growing the cells on media containing galactose and 5-FOA. For diploid SPI screens, the cells are kept as heterozygous diploids by growing them on synthetic glucose media. The screening is typically performed with 1536 colonies (96–384 strains with 4–16 replicates) arrayed on rectangular agar plates and a typical SPI screen takes a week to perform. (B) Venn diagram of SPI screens with GBP-tagged kinetochore proteins in haploid and heterozygous diploid GFP strains shows an overlap of 119 strain or ~50%. (C) The overlap of the 119 kinetochore SPIs found in inner and outer kinetochore SPI screens is shown. (D) Venn diagram showing outer kinetochore SPIs detected in both haploid and diploid GFP strains. Haploid-specific SPIs were excluded from this diagram and structural kinetochore proteins were also removed to highlight candidates of kinetochore regulation. Excluding the haploid-specific SPIs may omit interactions that affect kinetochore function; however, it also excludes growth effects caused by mislocalization of the GFP protein and so provides a conservative list of candidate kinetochore regulators. The Cnn1 is a subunit of the CCAN and thus should be technically considered an inner kinetochore protein, but it extends towards the outer kinetochore and many of the SPIs found in the Cnn1 screen overlap with outer kinetochore SPIs. ^ refers to GFP strains that were found as haploid and diploid SPIs with GBP-Cnn1 in contrast to Cnn1-GBP. Asterisk * refers to GFP strains that were also detected as haploid and dipl [file pgen.1008990.s003.tif]

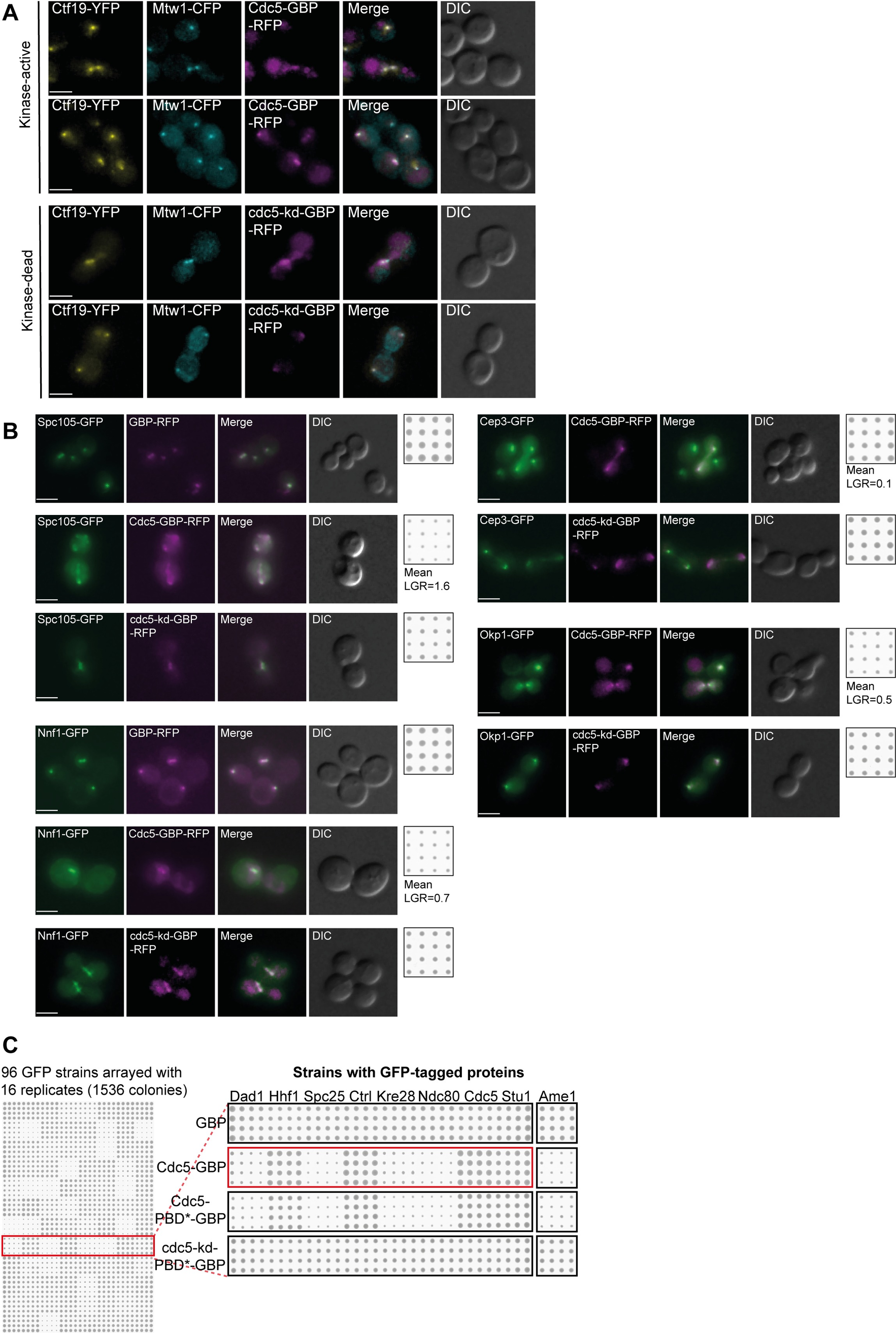

Supplement: S2 Fig — (A) Fluorescence microscopy with Ctf19-YFP (which binds GBP) and Mtw1-CFP (which does not bind GBP) to confirm that Cdc5-GBP and cdc5-kd-GBP are recruited to the kinetochore foci. (B) Examples of Cdc5-GBP recruitment to GFP-tagged kinetochore proteins. The resulting colonies from the SPI screen and the effect on growth indicated by log growth ratios (LGR) are shown on the right of the images for reference. All scale bars are 5μm. (C) Example of data from the Cdc5 kinetochore SPI screen showing each GFP strain arrayed with 16 replicates (in total 1536 colonies per plate). A cropped selection of GFP strains are shown on the right with Cdc5-GBP SPIs highlighted in red. (TIF) [file pgen.1008990.s004.tif]

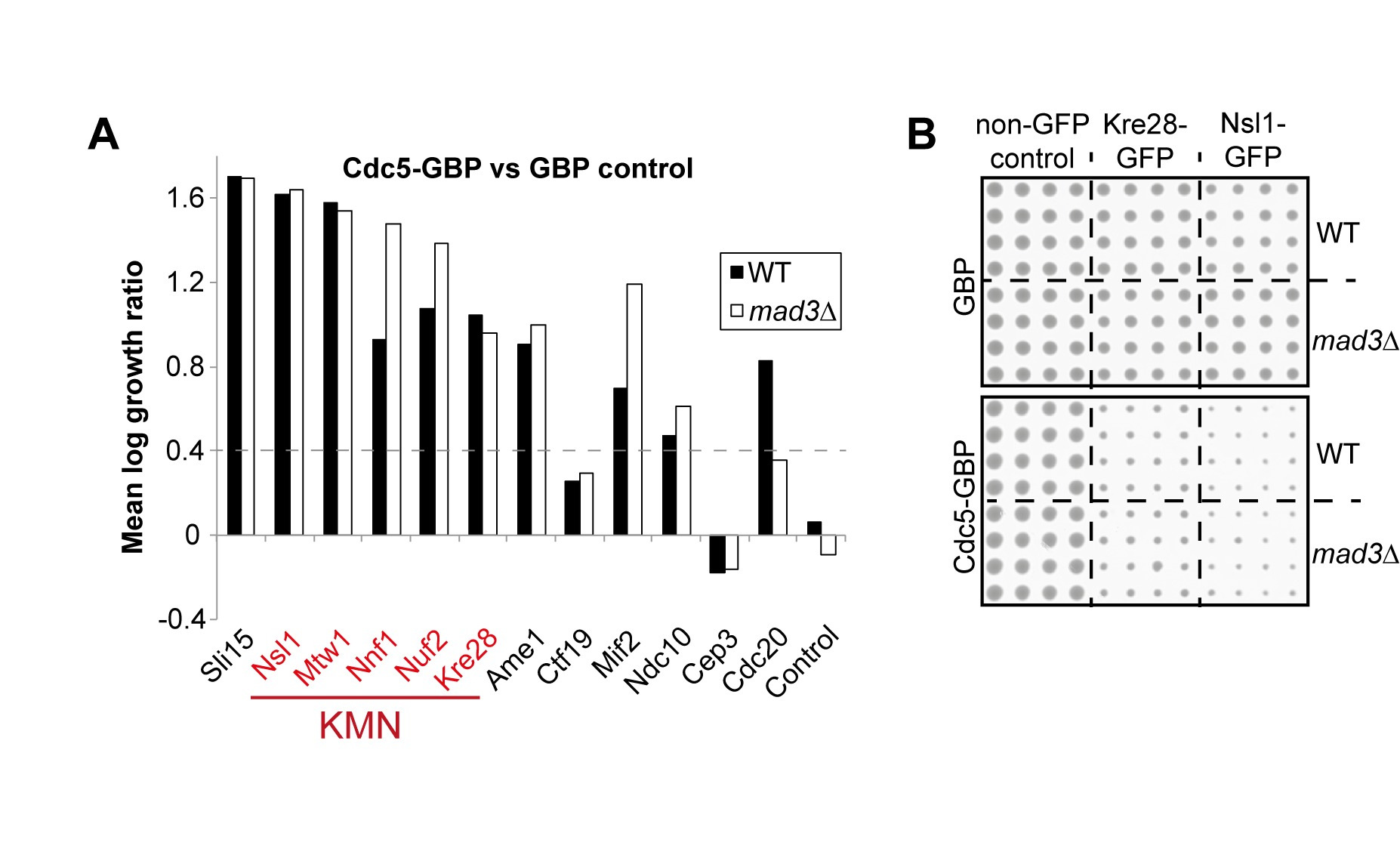

Supplement: S3 Fig — (A) To assess the SAC, the Cdc5 SPI screen was repeated with a selection of GFP-tagged kinetochore strains in both wild-type and mad3Δ cells. Deletion of MAD3 gene was not sufficient to suppress any Cdc5 kinetochore SPI except Cdc20-GFP. (B) Example of colonies from the Cdc5 kinetochore SPI screen with wild-type and mad3Δ GFP strains. (TIF) [file pgen.1008990.s005.tif]

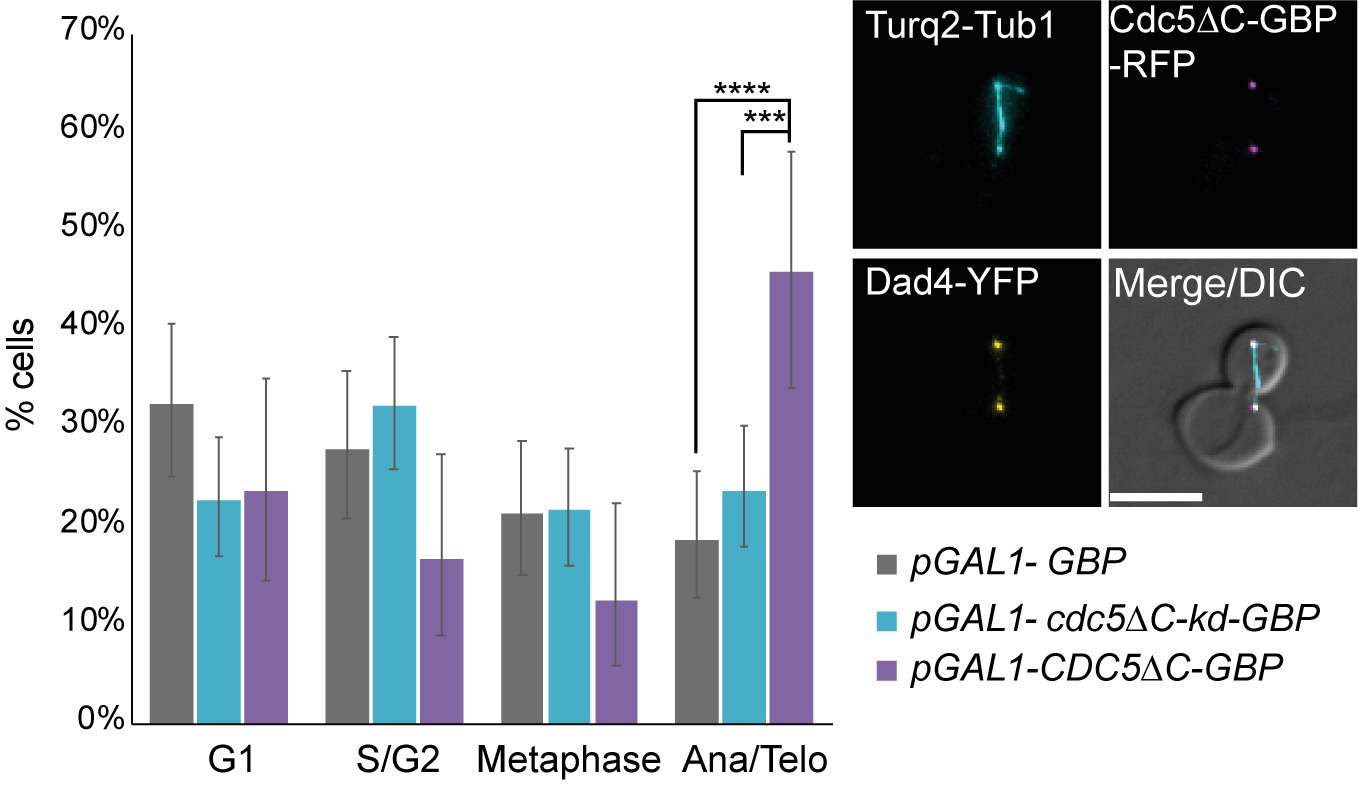

Supplement: S4 Fig — Asynchronous cultures of Dad4-YFP Turq2-Tub1 cells (T621) expressing CDC5ΔC-GBP, cdc5ΔC-kd-GBP or GBP alone, all under the control of GAL1 promoter were analyzed using fluorescence microscopy as in Fig 3C. Cells expressing CDC5ΔC-GBP (n = 144) are significantly increased in anaphase/telophase compared to cdc5ΔC-kd-GBP (n = 199) or GBP (n = 151) cells. Fishers exact test; p-values *** = p < 10−3, **** = p < 10−4. Error bars indicate 95% binomial C.I. The inset on the right shows a representative image of Dad4-YFP cells expressing Cdc5ΔC-GBP in anaphase. Scale bar is 5μm. (TIF) [file pgen.1008990.s006.tif]

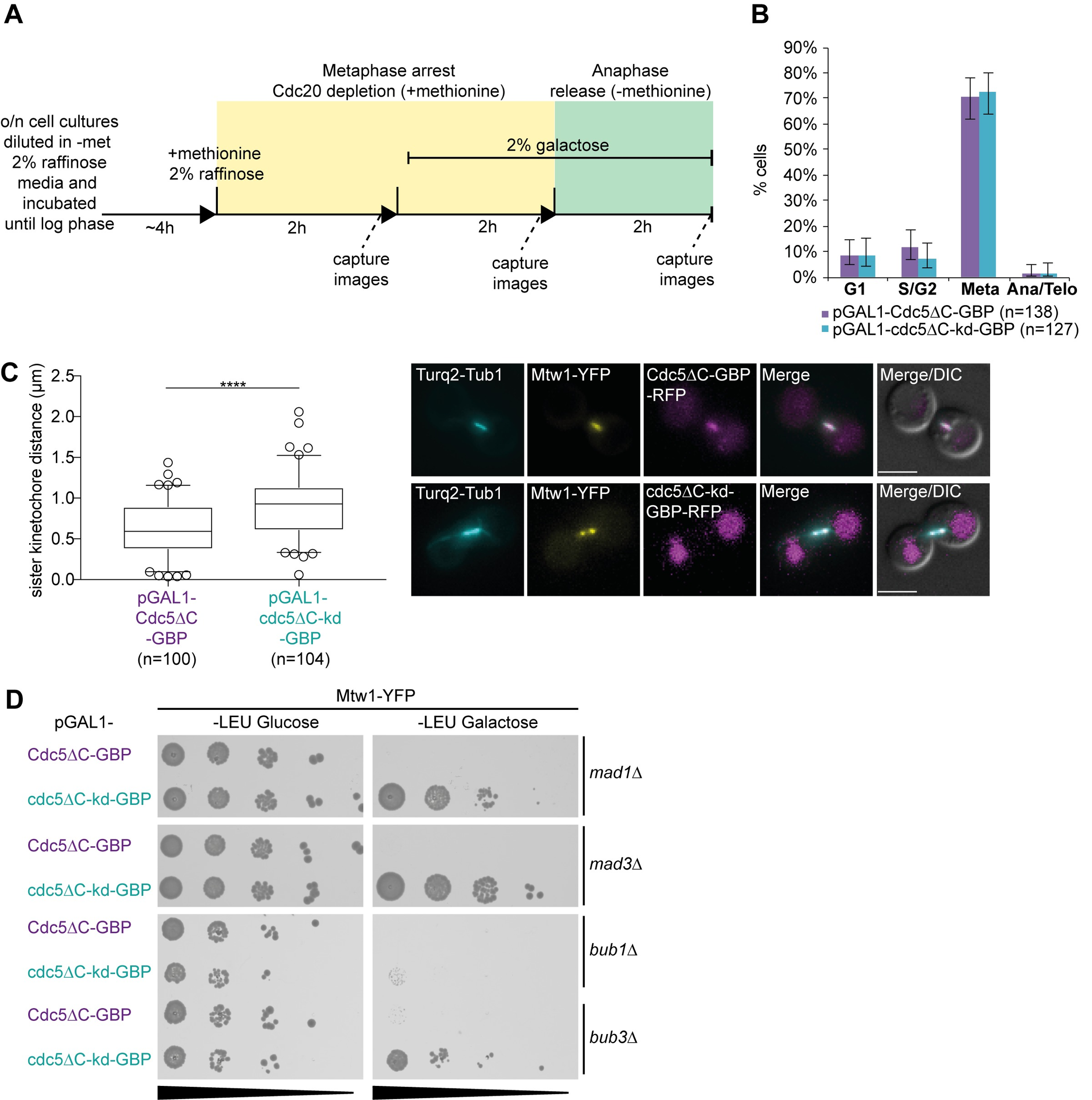

Supplement: S5 Fig — (A) Diagram describing the experimental setup of the metaphase-arrest and release analysis. See text and methods for further details. (B) Mtw1-YFP Turq2-Tub1 cells were arrested in metaphase by incubation in media containing methionine (Cdc20 depletion). After two hours ~70% of cells were arrested in metaphase. Error bars indicate 95% binomial C.I. (C) After two hours of galactose induction of either CDC5ΔC-GBP or cdc5ΔC-kd-GBP in the metaphase-arrested cells the distance between two sister kinetochores was measured using a semi-automated quantification tool (see Materials and methods for details). The box and whiskers plot indicates the mean sister kinetochore distance and standard deviation of the variance (line and box, respectively). The whiskers indicate the 95 percentile and outliers are indicated as circles. Statistical analysis was done using two-tailed student’s t-test; p-value *** = 5.4 x 10^−8^. Representative images are shown on the right. Scale bars are 5μm. (D) 10-fold serial dilutions spot assay with Mtw1-YFP (mad1Δ, mad3Δ, bub1Δ and bub3Δ) cells expressing either CDC5ΔC-GBP or cdc5ΔC-kd-GBP shows that the growth defect caused by the Cdc5-Mtw1 interaction is independent of the SAC. Interestingly expression of the cdc5ΔC-kd-GBP control became lethal in a bub1Δ strain. (TIF) [file pgen.1008990.s007.tif]

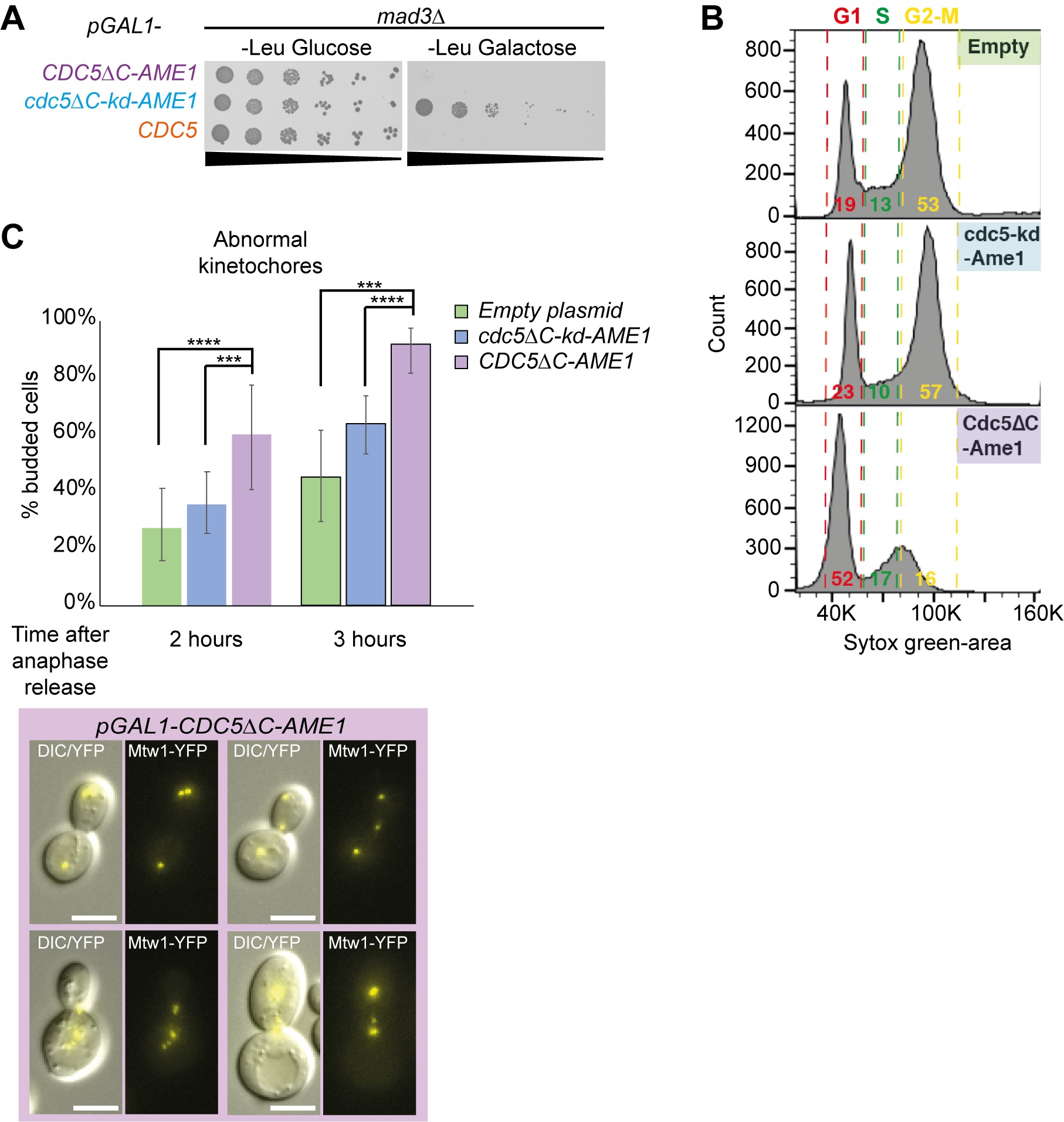

Supplement: S6 Fig — (A) 10-fold serial dilutions spot assay with mad3Δ strain expressing CDC5ΔC-AME1, cdc5ΔC-kd-AME1 or CDC5 alone shows that the growth defect caused by CDC5ΔC-AME1 expression is not dependent on the SAC. (B) Cell-cycle analysis by flow cytometry of cells expressing CDC5ΔC-AME1. Asynchronous log-phase cell cultures were grown in galactose for four hours before cell fixing and DNA staining with Sytox green and then measured with flow cytometry. The same G1 (red), S (green) and G2-M (yellow) gates were used for all samples. The colored numbers indicate the percentages of cells in each cell-cycle stage. This experiment was done in duplicate with identical results. See methods for further details. (C) Related to Fig 4F. After three hours of release from metaphase arrest, the majority of CDC5ΔC-AME1 expressing cells displayed a severe mitotic spindle phenotype. Budded cells with two or more Mtw1-YFP foci from the analysis in Fig 4F were reanalyzed and cells with more than two foci, fractured or abnormal Mtw1-YFP signal were categorized as abnormal mitotic spindles. After both two (non-outlined bars) and three (black-outlined bars) hours CDC5ΔC-AME1 expressing cells (n = 91 and n = 59) were significantly increased for abnormal kinetochores compared to cdc5ΔC-kd-AME1 (n = 82 and n = 40) and empty plasmid control (n = 52 and n = 30). Fishers exact statistical test; p-values *** = p < 10−3, **** = p < 10−5. Error bars indicate 95% binomial C.I. Examples of cells expressing CDC5ΔC-AME1 containing abnormal kinetochore foci are shown on the bottom. All scale bars are 5μm. (TIF) [file pgen.1008990.s008.tif]

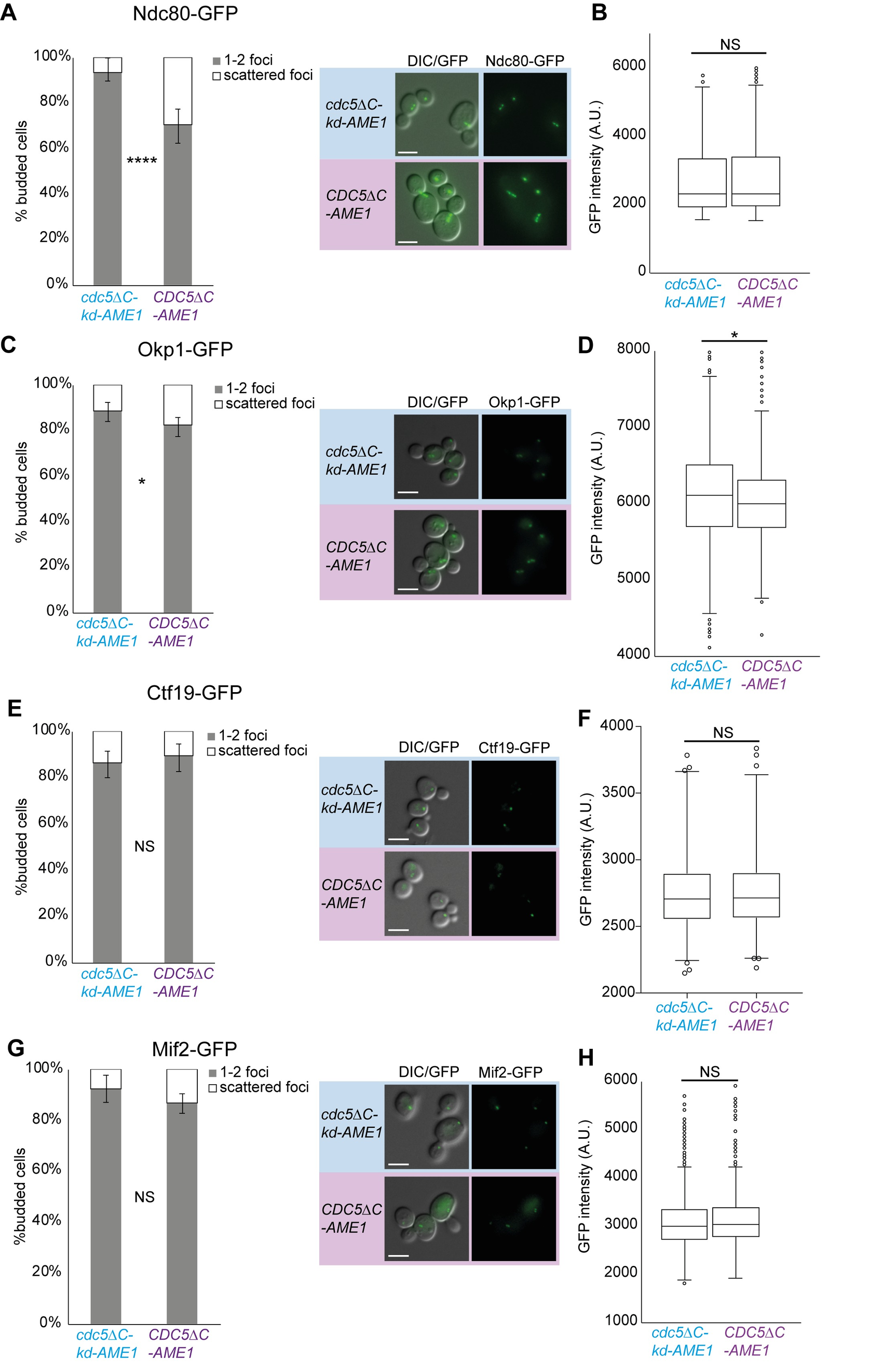

Supplement: S7 Fig — (A) Asynchronous cell cultures of Ndc80-GFP strain containing either pGAL1-CDC5ΔC-AME1 or pGAL1-cdc5ΔC-kd-AME1 plasmids were grown in galactose media for four hours before imaging with fluorescence microscopy. The Ndc80-GFP foci were analyzed and budded cells that exhibited declustered or scattered foci were quantified. Compared to cdc5ΔC-kd-AME1 control (n = 107) cells expressing CDC5ΔC-AME1 (n = 152) had significantly more cells with scattered Ndc80-GFP foci. Fishers exact test; p-values **** = p < 10−5. Error bars indicate 95% binomial C.I. Representative images are shown on the right. All scale bars are 5μm. (B) The Ndc80-GFP foci intensities in (A) are shown as a box and whiskers plot. Cells expressing CDC5ΔC-AME1 and cdc5ΔC-kd-AME1 were compared but no significant difference was found between the two. The mean Ndc80-GFP intensity and standard deviation of the variance are indicated with a line and box, respectively. The whiskers indicate the 95 percentile and outliers are indicated as circles. Statistical analysis was done using two-tailed student’s t-test. (C) Asynchronous cell cultures of Okp1-GFP strain containing either pGAL1-CDC5ΔC-AME1 or pGAL1-cdc5ΔC-kd-AME1 plasmids were analyzed in the same way as (A). Cells expressing CDC5ΔC-AME1 (n = 273) exhibited slightly more cells with scattered Okp1-GFP foci compared to cdc5ΔC-kd-AME1 expressing cells (n = 318). Fishers exact test; p-values * = p < 0.05. Representative images are shown on the right. (D) The Okp1-GFP foci intensities in (C) were compared between cells expressing CDC5ΔC-AME1 and cdc5ΔC-kd-AME1 as in (B). Cells expressing CDC5ΔC-AME1 had a slightly lower Okp1-GFP foci intensity compared to cdc5ΔC-kd-AME1 expressing cells. Statistical analysis was done using two-tailed student’s t-test; * = p < 10−4. (E) Asynchronous cell cultures of Ctf19-GFP strain containing either pGAL1-CDC5ΔC-AME1 (n = 167) or pGAL1-cdc5ΔC-kd-AME1 (n = 148) plasmids were analyzed in the same way as (A) and (C). There was no st [file pgen.1008990.s009.tif]

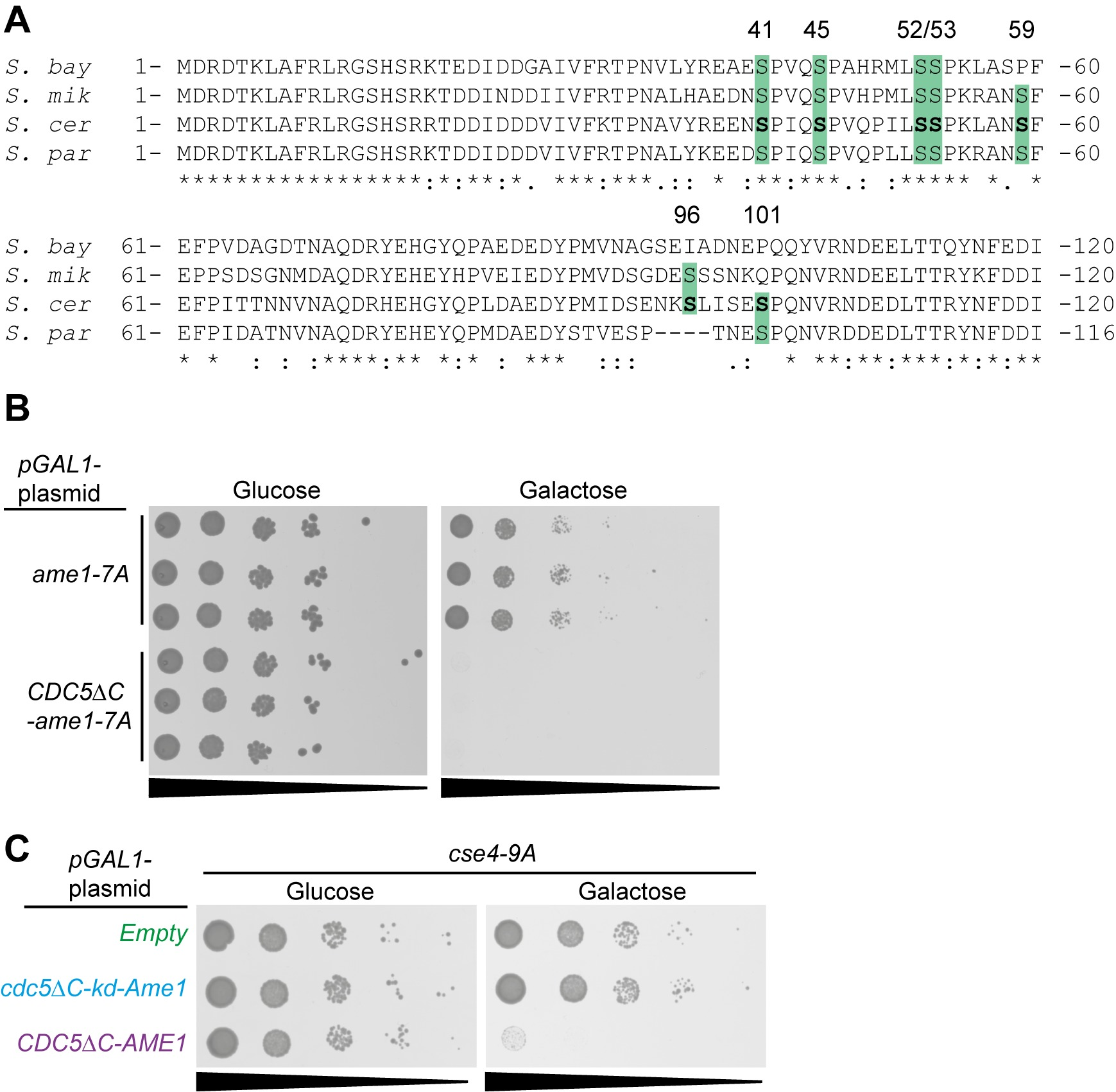

Supplement: S8 Fig — (A) The amino acid sequence of the N terminus of Ame1 and homologs of several related yeast species. Reported phosphorylation sites of Ame1 are shown in bold (thebiogrid.org). ClustalW alignment (www.ebi.ac.uk/Tools/msa/clustalo/) shows the evolutionary conservation of Ame1 N terminus among several yeast species. S. bay = Saccharomyces bayanus, S. mik = Saccharomyces mikatae, S. cer = Saccharomyces cerevisiae, S. par = Saccharomyces paradoxus. The seven phosphoserines within N-terminal Ame1 are highlighted green. (B) 10-fold serial dilutions spot assay with cells expressing ame1-7A control or CDC5ΔC-ame1-7A fusion (serines 41, 45, 52, 53, 59, 96 and 101 mutated to alanines) suggests that the growth defect caused by the Cdc5-Ame1 association is not dependent on N-terminal Ame1 phosphorylation. (C) 10-fold serial dilutions spot assay with cse4-9A strain expressing CDC5ΔC-AME1, cdc5ΔC-kd-AME1 or empty plasmid control suggests that the growth defect caused by CDC5ΔC-AME1 expression is not dependent on N-terminal Cse4 phosphorylation. (TIF) [file pgen.1008990.s010.tif]

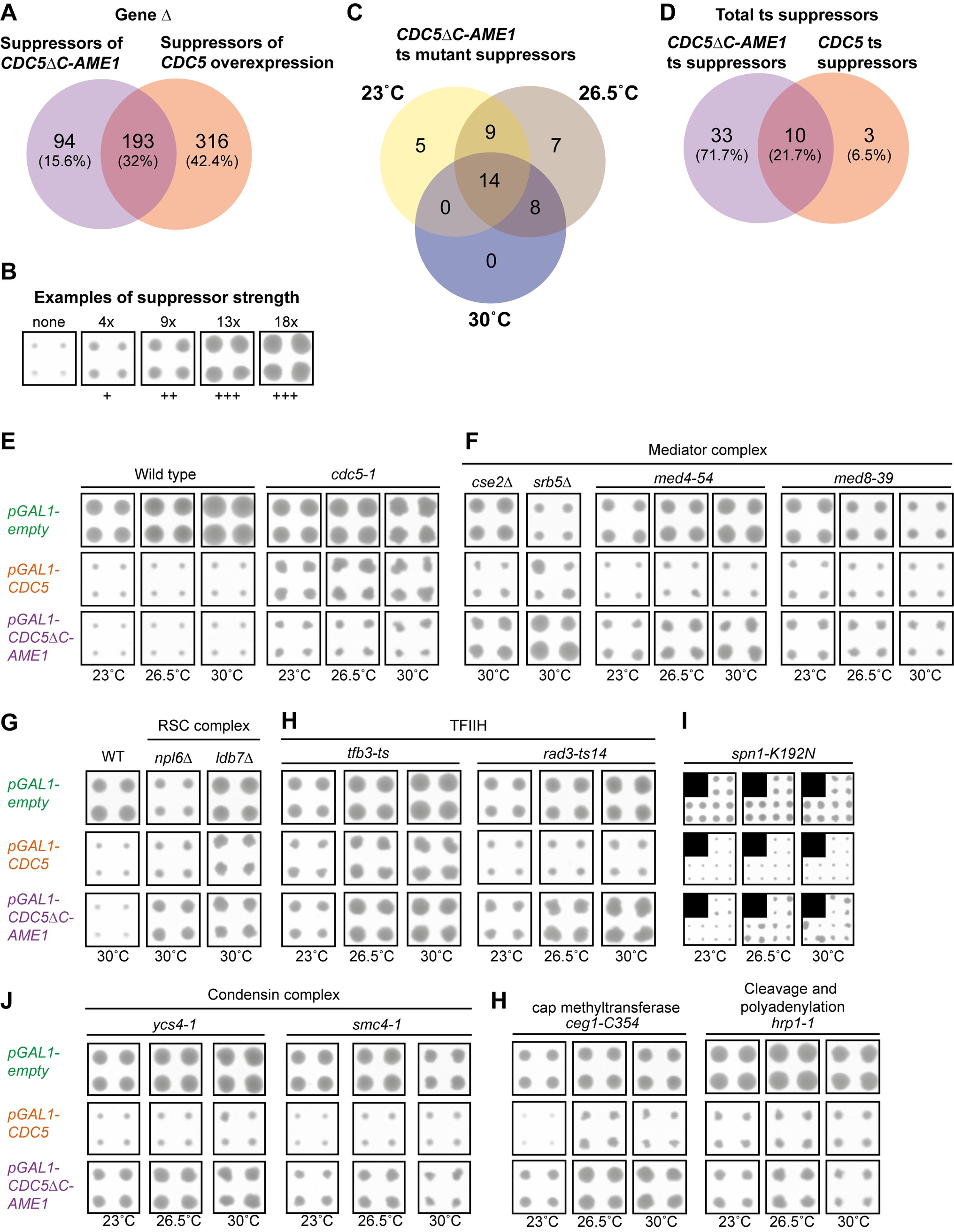

Supplement: S9 Fig — (A) Venn diagram showing the overlap between gene deletion (Δ) suppressors of CDC5ΔC-AME1 growth phenotype and suppressors of CDC5 overexpression. (B) Examples of the strength of suppression of the growth defects. Colonies that were four times as big as the plate median colony size was used as a cut-off for suppression. We estimate that as a conservative threshold since some plates had a higher frequency of larger colonies. We organize the data according to the strength of suppression; + weak, ++ moderate and +++ strong suppression compared to wild type. See S4 Data for all suppressor data and methods for further details. (C) Venn diagram showing the overlap of temperature-sensitive mutant (ts) suppressors of CDC5ΔC-AME1 growth phenotype at three different temperatures (23°C, 26.5°C and 30°C). In total 43 suppressors. (D) Venn diagram showing the overlap of temperature-sensitive mutant (ts) suppressors of CDC5ΔC-AME1 growth phenotype (43 in total) and CDC5 overexpression (13 in total). (E-H) A selection of cropped images of colonies from the suppressor screens. Mutants or deletions of components of the Mediator complex (F), the RSC complex (G), TFIIH (H), Spn1/Iws1 (I), the Condensin complex (J), and mRNA-processing (H) were found as suppressors of the growth defect caused by CDC5ΔC-AME1. (TIF) [file pgen.1008990.s011.tif]

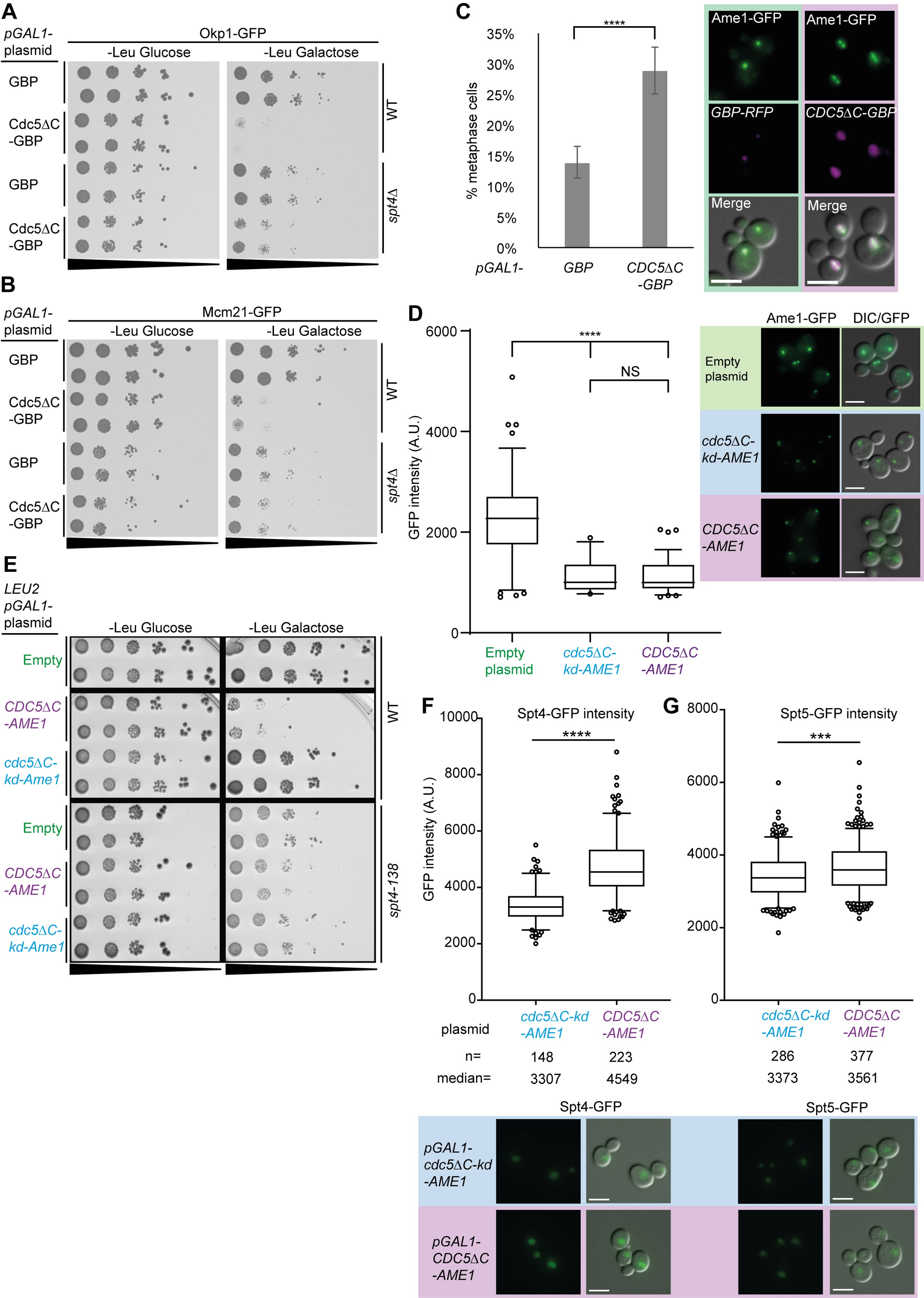

Supplement: S10 Fig — (A-B) 10-fold serial dilutions spot assay with wild-type and spt4Δ Okp1-GFP (A) and Mcm21-GFP (B) strains expressing CDC5ΔC-GBP or GBP control shows that the growth defect caused by the Cdc5-COMA associations depends on SPT4. (C) Analysis of Ame1-GFP cells, which were imaged with fluorescence microscopy after 4 hours of growth in 2% galactose media to induce expression of either pGAL1-CDC5ΔC-GBP (n = 566) or pGAL1-GBP (n = 718). Cells that did not show RFP/GFP colocalization were excluded from this analysis. Cells expressing Cdc5ΔC-GBP-RFP were significantly increased in metaphase compared to control. Fishers exact statistical test; p-values **** = p = 3.38 x 10^−11^. Error bars indicate 95% binomial C.I. Representative images are shown on the right. Scale bars are 5μm. (D) Ame1-GFP foci in cells expressing cdc5ΔC-kd-AME1 or CDC5ΔC-AME1 were analyzed with fluorescence microscopy and the data is shown as box and whiskers plots. The mean Ame1-GFP intensity and standard deviation of the variance are indicated with a line and box, respectively. The whiskers indicate the 95 percentile and outliers are indicated as circles. Asynchronous cell cultures of Ame1-GFP strain containing either an empty, pGAL1-cdc5ΔC-kd-AME1 or pGAL1-CDC5ΔC-AME1 plasmids were grown in galactose media for four hours before imaging. Compared to the empty plasmid control (n = 96), expression of either cdc5ΔC-kd-AME1 (n = 31) or CDC5ΔC-AME1 (n = 79) had significantly reduced Ame1-GFP signal. Two-tailed student’s t-test; p-values **** = p < 10−10. Representative images are shown on the right. Scale bars are 5μm. (E) 10-fold serial dilutions spot assay showing that a spt4-138 mutant can also rescue the growth defect caused by CDC5ΔC-AME1. (F-G) Spt4-GFP and Spt5-GFP fluorescence signals were analyzed after inducing either CDC5ΔC-AME1 or cdc5ΔC-kd-AME1 for four hours as in (D). Statistical analysis was done using two-tailed student’s t-test; **** = p <10−8, *** = p <10−4. Representative images are shown o [file pgen.1008990.s012.tif]

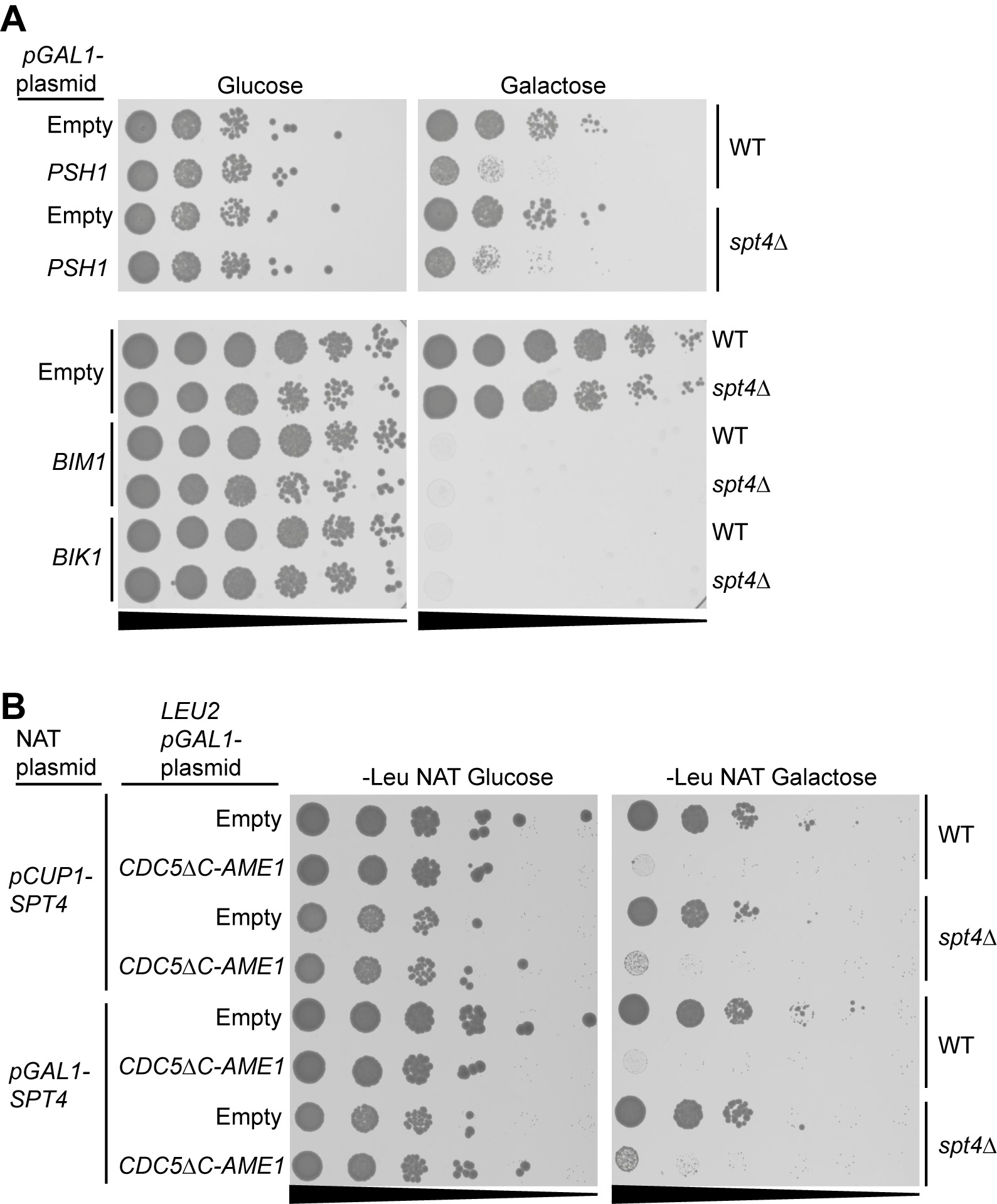

Supplement: S11 Fig — (A) 10-fold serial dilutions spot assay with wild-type and spt4Δ strains overexpressing PSH1 or empty control (top panel) and a 5-fold serial dilutions spot assay with wild-type and spt4Δ strains overexpressing BIM1, BIK1 or empty control (bottom panel). Deletion of SPT4 does not suppress the growth defect caused by the overexpression of any of the genes. (B) 10-fold serial dilutions spot assay with wild-type and spt4Δ strains expressing either CDC5ΔC-AME or empty control and coexpressing SPT4 driven by either a CUP1 promoter (top 4 rows) or GAL1 promoter (bottom 4 rows). In both cases the coexpression of SPT4 represses the spt4Δ-dependent rescue of the growth defect caused by CDC5ΔC-AME1. (TIF) [file pgen.1008990.s013.tif]

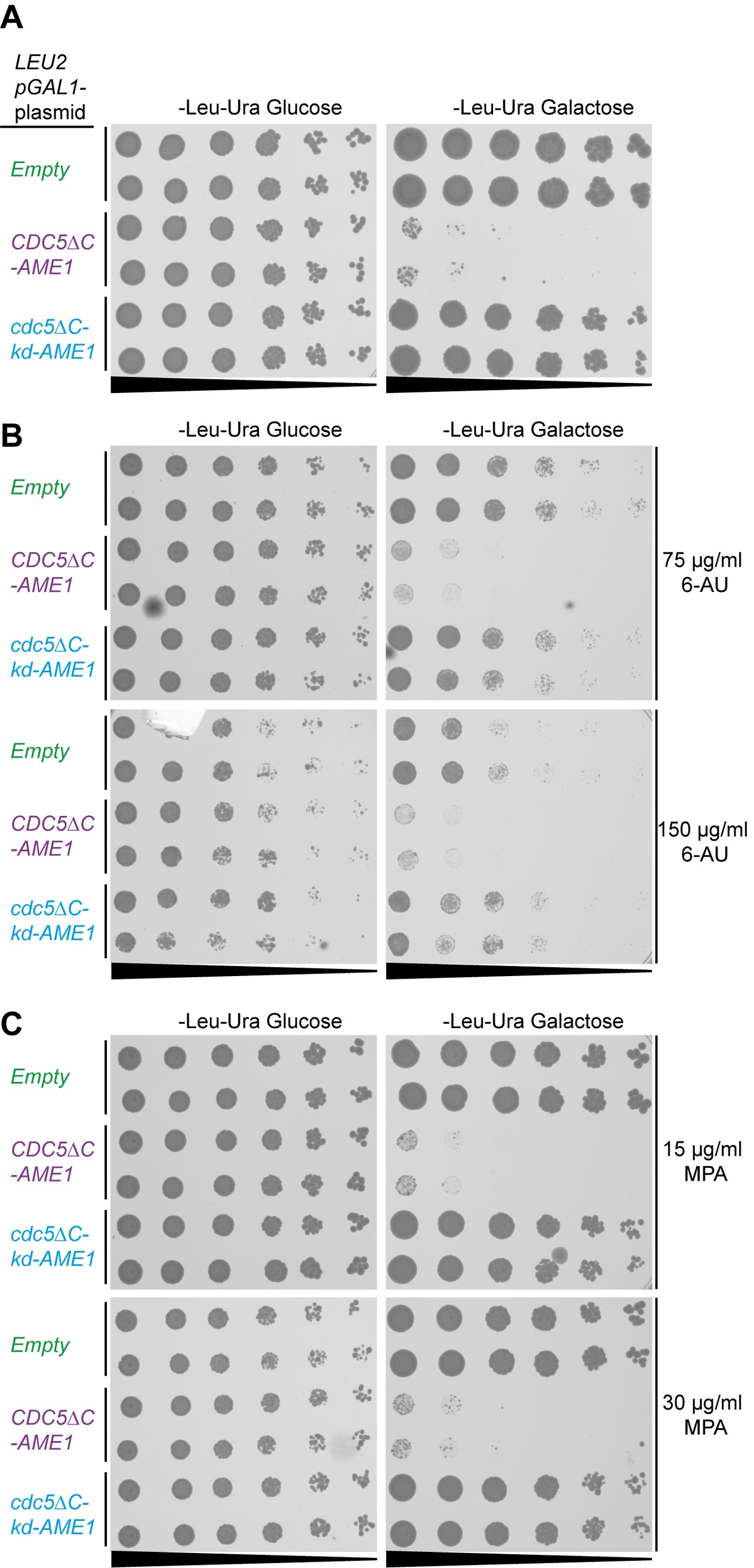

Supplement: S12 Fig — (A) 5-fold serial dilutions spot assay with wild-type cells expressing either CDC5ΔC-AME1, cdc5ΔC-kd-AME1 or empty plasmid. (B) Spot assay as in (A) was repeated with two concentrations of 6-Azauracil (6-AU) added to the media. The agar plates were incubated for two additional days (4 in total) at 30°C. (C) Spot assay as in (A) was repeated with two concentrations of mycophenolic acid (MPA) added to the media. The agar plates were incubated for two additional days (4 in total) at 30°C. (TIF) [file pgen.1008990.s014.tif]

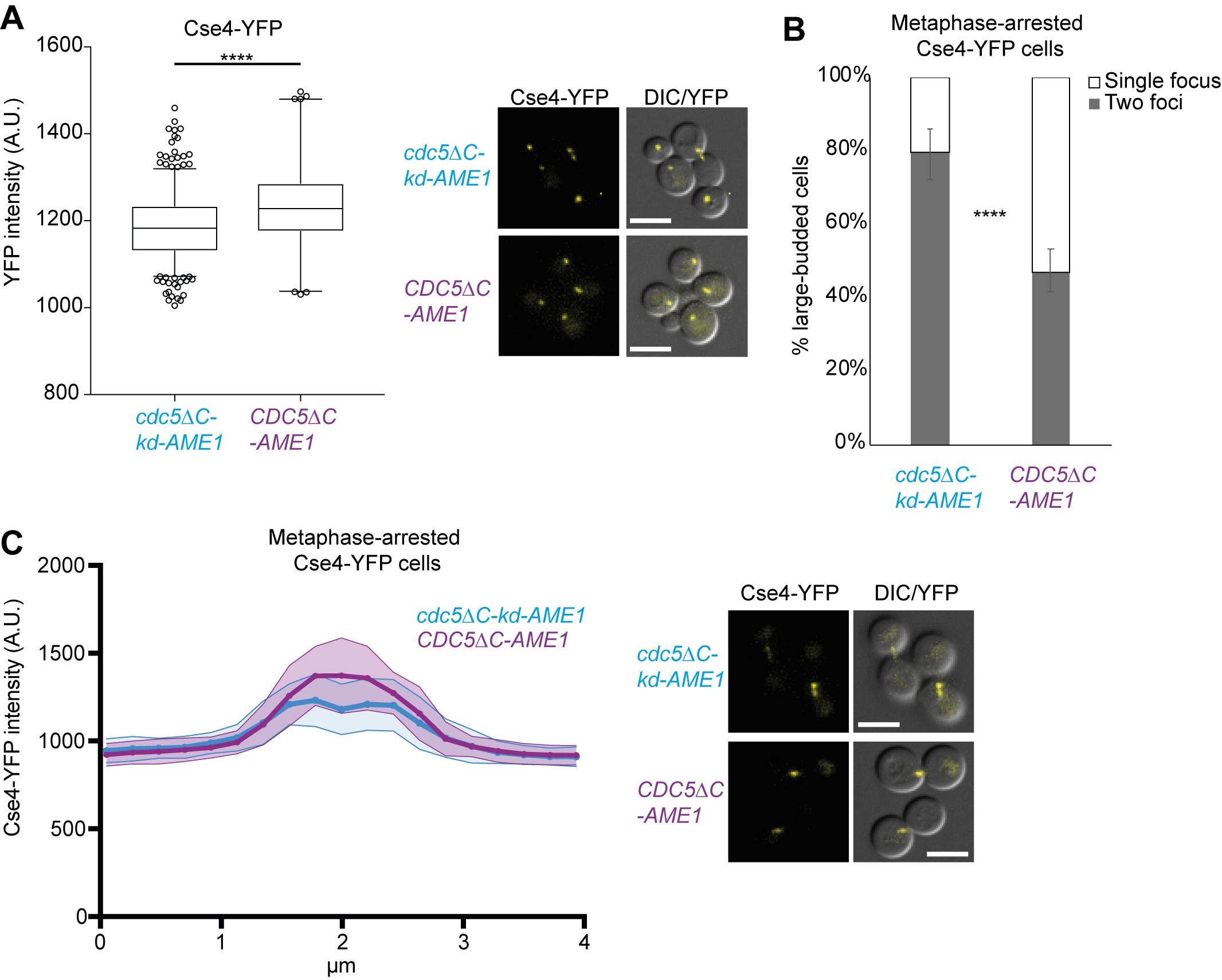

Supplement: S13 Fig — (A) Asynchronous cell cultures of Cse4-YFP (internally tagged) strain containing either pGAL1-CDC5ΔC-AME1 or pGAL1-cdc5ΔC-kd-AME1 plasmids were grown in galactose media for four hours before imaging with fluorescence microscopy. The Cse4-GFP foci intensity was quantified. Compared to cdc5ΔC-kd-AME1 control (n = 456) cells expressing CDC5ΔC-AME1 (n = 483) had significantly increased Cse4-YFP foci intensity. The mean Cse4-YFP intensity and standard deviation of the variance are indicated with a line and box, respectively. The whiskers indicate the 95 percentile and outliers are indicated as circles. Statistical analysis was done using two-tailed student’s t-test; **** = p <10−8. Representative images are shown on the right. Scale bars are 5μm. (B) Cse4-YFP (internally tagged) cells were arrested in metaphase using Cdc20 depletion and large-budded cells containing a single Cse4-YFP focus or two foci were quantified. Cells expressing CDC5ΔC-AME1 (n = 222) during metaphase-arrest had increased number of cells with a single Cse4-YFP focus compared to cells expressing cdc5ΔC-kd-AME1 (n = 161). Fishers exact test; p-values **** = p < 10−10. Error bars indicate 95% binomial C.I. (C) The Cse4-YFP signal from cells in (B) was measured along the mitotic spindle after inducing CDC5ΔC-AME1 or controls for two hours (see S5A Fig for further description of experimental setup). A 4 μm line with 19 points was used to include background signal and to cover the spread signal phenotype in cells expressing CDC5ΔC-AME1 (40 randomly selected mitotic spindles were measured for each condition). The shadowed area indicates standard deviation. Representative images are shown on the right. Scale bars are 5μm. (TIF) [file pgen.1008990.s015.tif]

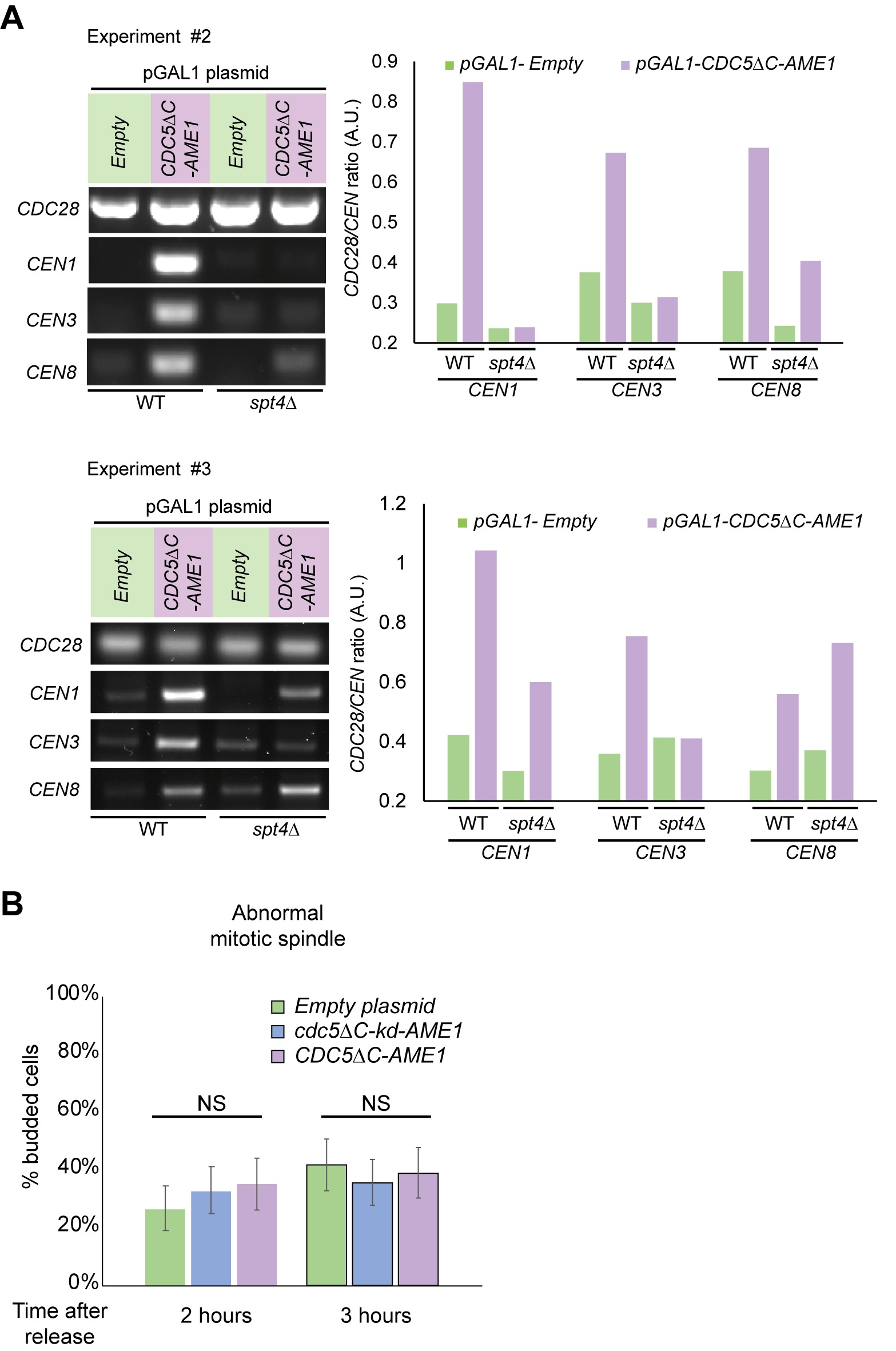

Supplement: S14 Fig — (A) Reverse transcription PCR (RT-PCR) experiments were repeated as in Fig 6A. Quantification of the relative CEN RNA levels is shown in the graph on the right. Ratio of the CDC28 RNA control to the CEN RNAs was calculated. (B) Cells from the analysis in Fig 6B were released into anaphase and analyzed after two (non-outlined bars) and three (black-outlined bars) hours as in S6C Fig. The abnormal kinetochore phenotype seen in Fig 4F and S6C Fig was reduced by spt4Δ and there was no significant difference between spt4Δ cells expressing CDC5ΔC-AME1 (n = 127 and n = 132), cdc5ΔC-kd-AME1 (n = 141 and n = 150) or empty plasmid controls (n = 137 and n = 132). Fishers exact statistical test. Error bars indicate 95% binomial C.I. (TIF) [file pgen.1008990.s016.tif]

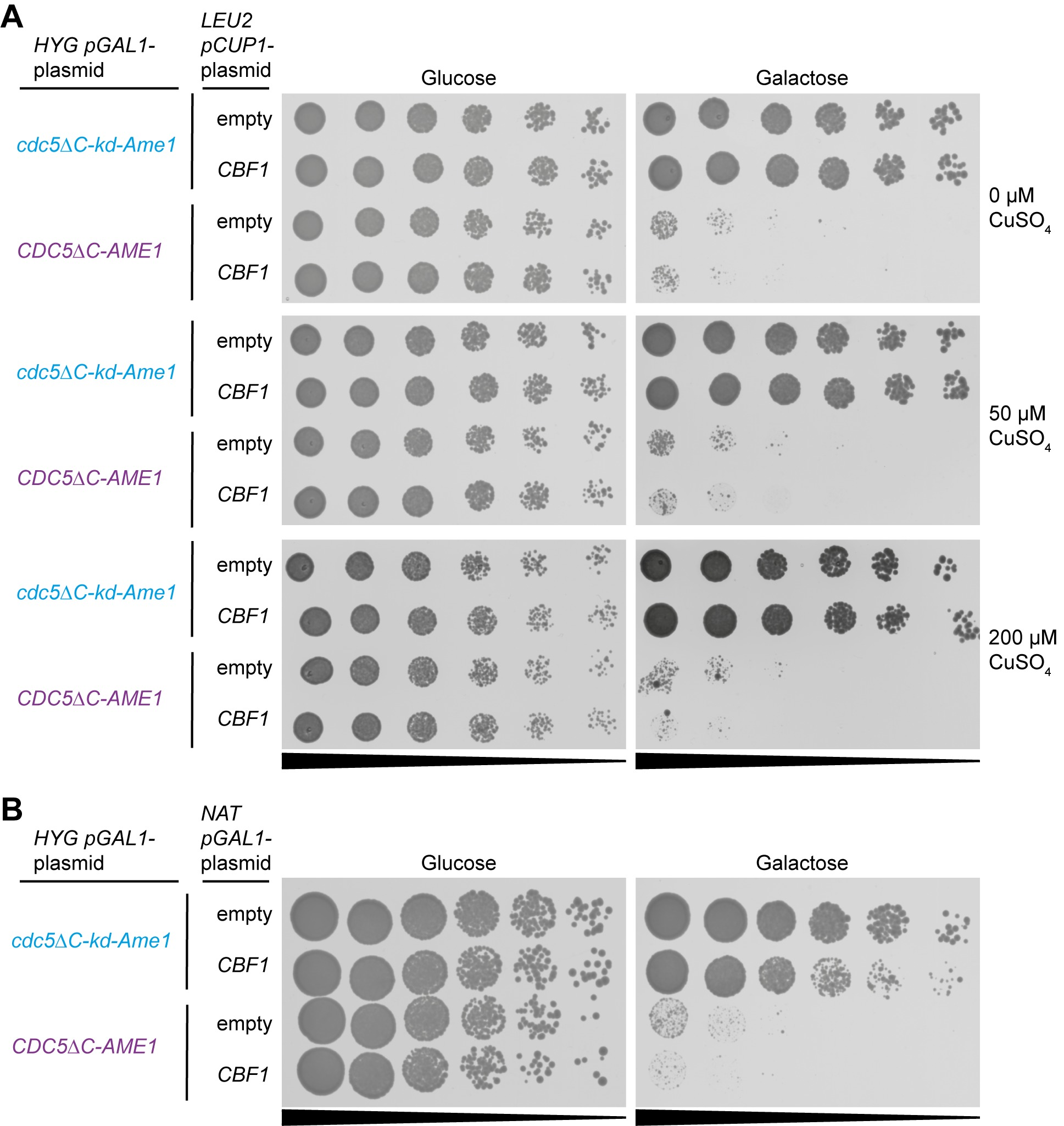

Supplement: S15 Fig — (A) 5-fold serial dilutions spot assay with cells expressing either CDC5ΔC-AME or cdc5ΔC-kd-AME1 and coexpressing either a pCUP1-driven CBF1 or an empty plasmid control with either no copper (CuSO4) added to the media for low expression of CBF1, or for increased CBF1 expression 50μM or 200μM was added. In none of the cases was overexpression sufficient to suppress the growth defect caused by CDC5ΔC-AME expression. (B) 5-fold serial dilutions spot assay with cells expressing either CDC5ΔC-AME or cdc5ΔC-kd-AME1 and either overexpressing CBF1 using the GAL1 promoter or an empty plasmid control. Overexpression of CBF1 driven by pGAL1 was insufficient to suppress the CDC5ΔC-AME growth phenotype. (TIF) [file pgen.1008990.s017.tif]
