# Supplementary material for: Polo kinase recruitment via the constitutive centromere-associated network at the kinetochore elevates centromeric RNA
Source: PLoS Genet. 2020 Aug 18;16(8):e1008990. doi: 10.1371/journal.pgen.1008990 (PMC7455000; doi:10.1371/journal.pgen.1008990)
Supplement: Attachment — Submitted filename: Response to Reviewers Olafsson.docx [file pgen.1008990.s022.docx]

Response to Reviewers Olafsson & Thorpe.

Editor and reviewers comments are in black text, our responses are in blue text and changes to the text indicated with red text.

Dear Dr Thorpe,

Thank you very much for submitting your Research Article entitled 'Polo kinase recruitment via the constitutive centromere-associated network at the kinetochore elevates centromeric RNA.' to PLOS Genetics. Your manuscript was fully evaluated at the editorial level and by three independent peer reviewers. The reviewers all agreed that the study was interesting, focused an important topic, and provided important new data to the field, however, they identified a few aspects of the manuscript that should be improved.

We therefore ask you to modify the manuscript according to the review recommendations before we can consider your manuscript for acceptance. Your revisions should address the specific points made by each reviewer. We understand that it might not be feasible to address all comments experimentally, but encourage the incorporation of any additional existing data that addresses the reviewers' concerns. The revision should also include:

a. clarification or simplification of  the SPI screening approach to educate and aid readers who may be less familiar with this technique (as noted by all reviewers)

See our response to point 2 of reviewer #1.

b. incorporation of error bars, statistical measures, for multiple figures (as noted by several reviewers)

See our responses to reviewer #3.

c. clearer rationale for the focus on Cdc5 considering the other hits/interactions that were revealed by the screen

See our response to point 1 of reviewer #1.

d. additional explanation or elaboration on the growth arrest phenotype

See our response to point 17 of reviewer #2.

e. insight into the relationship between Ame1 and Cdc5 (see R2 comments)

See our response to point 2 of reviewer #2.

In addition we ask that you:

1) Provide a detailed list of your responses to the review comments and a description of the changes you have made in the manuscript.

2) Upload a Striking Image with a corresponding caption to accompany your manuscript if one is available (either a new image or an existing one from within your manuscript). If this image is judged to be suitable, it may be featured on our website. Images should ideally be high resolution, eye-catching, single panel square images. For examples, please browse our [archive](https://eur01.safelinks.protection.outlook.com/?url=http%3A%2F%2Fwww.plosgenetics.org%2Farticle%2Fbrowse%2Fvolume&data=02%7C01%7C%7C1e2418be0e074647fb5408d8114af7a1%7C569df091b01340e386eebd9cb9e25814%7C0%7C0%7C637278360972453681&sdata=IGqNEw4TqdwG1SsiiTISrH3yGNt9O2XHe5wd72lHVps%3D&reserved=0). If your image is from someone other than yourself, please ensure that the artist has read and agreed to the terms and conditions of the Creative Commons Attribution License. Note: we cannot publish copyrighted images.

Reviewer #1: This manuscripts employs Synthetic Physical Interactions (SPIs) screens, mutagenesis and growth assays in yeast to investigate the interactions between kinases and phosphatases and the kinetochore focusing in particular on the Polo kinase/Cdc5. The ‘systems’ approach taken by this work, allows the authors to build on known and novel interactions to shed light into the complex regulation of kinetochore proteins by phosphorylation. Importantly, this work reveals a possible role for Polo kinase in regulating CEN transcription.

By forcing the recruitment of Cdc5 to different kinetochore subcomplexes, the authors identify multiple effects that are consistent with the many roles that Polo kinase plays at the kinetochore. Interestingly, phenotypes resulting from the constitutive recruitment of Cdc5 to the COMA complex are rescued by mutations in genes involved in chromatin remodeling and transcriptional elongation. One of these phenotypes is an increase in CEN RNA levels and this phenotype is dependent on the transcriptional regulator Spt4. Recruitment of proteins involved in transcription to the kinetochore also cause caused growth phenotypes, suggesting that perturbing normal CEN RNA level is deleterious for cell cycle progression.

Cdc5 could be influencing CEN transcription either directly or indirectly and, given the SPI interactions between Cdc5 and transcriptional regulators, the authors speculate that the role of Cdc5 may be direct, such as via the phosphorylation of RNAP II.

I only have a couple suggestions below.

1) I struggled connecting figure 1 (five SPIs for kinetochore proteins three of which were previously published) with the rest of the study, which focuses on Cdc5. Making this connection clearer would help the reader.

We have modified the results section to address this concern. See responses to comment 12) from reviewer #2 and the first comment from reviewer #3.

Regarding the additional SPI screens, we feel that the extensive SPI screening layed out in Figure 1 and S1 Fig was beneficial for the following reasons: First, previous SPI screens were done with outer kinetochore subunits not inner. Second, in order to identify novel regulators of the kinetochore we felt that additional screening would be beneficial for a high quality dataset that would help us decide which candidate to follow up. Third, the additional SPI screening was useful to compare kinetochore SPIs produced by different phosphatases and kinases. Fourth, the additional kinetochore SPI screens were required for a meaningful cluster analysis which showed that the Cdc5 phenotype is distinct from other kinases. We address these points in the introduction and the results:

Introduction:

“In this study we focused on Cdc5. First, we compared the forced recruitment of Cdc5 with other kinases and candidates of kinetochore regulation by probing the entire kinetochore using the SPI system. We identified many proteins, including Cdc5, that perturb kinetochore function, some of which have known kinetochore roles whereas others have no known kinetochore function. Second, we performed a proteome-wide SPI screen with Cdc5 and forcibly recruited it to every GFP-tagged protein. We found that Cdc5 SPIs were enriched for kinetochore, RNA polymerase II transcription factor complex (RNAPII) and SAGA histone acetyltransferase complex proteins.”

We now also point this out in the result section, for example, when we summarise the findings shown in Fig 1 and S1 Fig:

“Taken together the kinetochore SPI data highlight three key points. First, that the effect of recruiting candidate regulators is specific for the individual subcomplexes within the kinetochore (S1C-D Figs). For instance, most of the HDAC proteins were specifically identified in the Mtw1 and Ctf3 SPI screens. Second, that both kinases and phosphatases cause kinetochore SPI phenotypes, suggesting that constitutive phosphorylation or dephosphorylation of kinetochore subcomplexes is detrimental for cell growth. Third, the mitotic kinase, Cdc5, stands out as producing phenotypes across the kinetochore - in particular with Mif2, Mtw1 and Nuf2 - consistent with multiple functions at distinct kinetochore subcomplexes.  Furthermore, the Cdc5 SPI phenotype is most similar to that of the key cell-cycle phosphatase Cdc14 (Fig 1C, purple group). Therefore, we wanted to determine how a misregulated Cdc5 activity at different kinetochore subcomplexes is perturbing kinetochore function.”

2) I’m not familiar with the SPI system, could the authors include a conceptual summary of the approach when introducing the screen in Figure 1A, so the reader does not need to read previous papers to understand what’s being done here? The illustration is clear in terms of what the approach consists of, but clearly stating what biological information is gained by forcing proteins to come together would be helpful. What is the meaning of a slower growth when 2 proteins are forced together? Being such an artificial system, its advantage is not immediately apparent. It’s clearly an established and valid approach used routinely in other papers from this lab, it just needs a little bit of explanation.

We have modified supplementary figure 1 now displaying a schematic of the SPI methodology (S1A Fig) and added the following description of the SPI system in the figure legends and beginning of the results:

Legend to Fig S1...

“**S1 Fig. Kinetochore Synthetic Physical Interaction (SPI) screens.** (A) A schematic of the SPI method. A plasmid containing a gene of interest fused to GBP (e.g. *MTW1-GBP*) or control plasmids are introduced into a universal donor strain (UDS), containing a *URA3* locus and a conditional centromere on every chromosome. The UDS is mated with arrayed GFP strains, generating heterozygous diploid strains. For haploid SPI screens, the selective ploidy ablation technique is used to destabilize and select against the UDS chromosomes by growing the cells on media containing galactose and 5-FOA. For diploid SPI screens, the cells are kept as heterozygous diploids by growing them on synthetic galactose media. The screening is typically performed with 1536 colonies (96-384 strains with 4-16 replicates) arrayed on rectangular agar plates and a typical SPI screen takes a week to perform.”

Results....

“Synthetic Physical Interactions (SPIs) have been successful in identifying proteins that regulate the kinetochore [18,20]. The SPI system (Fig 1A, bottom panel, and S1A Fig), which utilizes the GFP-binding protein (GBP; also known as a nanobody [32]) and the genome-wide GFP collection [33], allows us to artificially associate two proteins in live cells, which for most pairs does not affect cell growth [20]. However, for specific complexes within the cell, such as the kinetochore, the recruitment of regulator proteins is carefully controlled such that they act at a specific time during the cell cycle or under specific circumstances. For example, if kinetochores dissociate from the spindle microtubules, proteins are recruited to the kinetochore to activate the spindle assembly checkpoint. The rationale for using SPIs is that this spatiotemporal control can be disrupted with constitutive protein associations. Thus, a regulator is locked in place, regardless of cell cycle stage or circumstances. Since kinetochore function is essential, such mis-regulation is likely to result in a growth defect. Consistent with this notion, artificial associations of characterised regulators to the kinetochore perturbs growth [13,18,22,34,35].”

Reviewer #2: Title: Polo kinase recruitment via the constitutive centromere-associated network at the

kinetochore elevates centromeric RNA.

Authors: Guðjón Ólafsson and Peter H. Thorpe

Understanding the regulation of kinetochore function is an important area of research because of its significance to cancer biology. Despite this, we have limited understanding of structural and functional architecture of the kinetochores. In this manuscript, authors have used high-through put synthetic protein interaction (SPI) approach to identify genes involved in the regulation of kinetochore function in budding yeast. In this study, authors performed two SPI screens and compared these results with the data from their three previously published screens. The SPI screens showed enrichment of proteins involved largely in post-translational modifications for example, protein kinases, phosphatases and chromatin modifiers. They performed in-depth investigation on effects of constitutive Cdc5 kinase activity and identified kinetochore as a major target where targeting Cdc5 to different kinetochore components resulted in phenotypes that were consistent with the role of different subcomplexes of the kinetochore. Authors suggest that constitutive Cdc5 kinase activity at kinetochore is mediated by centromeric RNA via Spt4-regulated process.

The manuscript is very well-written, nicely organized into sections, however, it requires simplification for the broad audience of the journal. The objectives of this study are logical, broad, hypothesis-driven, and thought-provoking. The results presented are experimentally sound, constitute novel addition to the literature in the field, and provide deeper research insights into the current state of knowledge in centromere biology and chromosome segregation.

Specific comments are below:

1) The authors should give a bit more introduction to SPI as this is the crux of the whole paper.

See response to comment 2) from reviewer #1. We have modified supplementary figure 1 now displaying a schematic of the SPI methodology (S1A Fig) and added further description of the SPI system in the figure legends and beginning of the results.

2) Authors have utilized a fusion construct of Cdc5 and Ame1 to examine the effect of constitutive association of Cdc5 with kinetochores. However, their previous studies have used independent constructs (Cdc5-GBP and kinetochore proteins tagged with GFP), which were forced to interact in-vivo upon their induction. The caveat in the fusion construct is that it does not allow to differentiate the contribution of individual proteins to the phenotypes observed in this study. Is it unclear why authors have not tried tagging the N-terminus of Ame1 with the GFP as they have done for other kinetochore proteins in their previous studies? Does Ame1-GFP and Cdc5-GBP exhibit SPI phenotype.

In this study we have used both direct fusions and the GFP-GBP system to induce Cdc5 association with Ame1. Cdc5-GBP expression in cells containing Ame1-GFP inhibits growth (which is rescued by deletion of *SPT4*); see Fig 5E, consistent with the direct *CDC5∆C-AME1* fusion. We agree that it would be worth tagging Ame1 N-terminally with GFP in order to place Cdc5∆C-GBP at the N terminus. However, Polo kinase (without the PBD) has been tethered to the N terminus of a number of proteins using direct genetic N-terminal fusions in both yeast and human cells;  therefore we decided to use the same approach. Additionally, since *spt4∆* rescued both the *CDC5∆C-AME1* fusion and Cdc5-GBP in Ame1-GFP, Okp1-GFP and Mcm21-GFP cells, suggests that the precise localisation of Cdc5 at the COMA complex is not crucial for the phenotype. Moreover, the direct *CDC5∆C-AME1* fusion plasmid was also utilized to introduce the forced Cdc5-Ame1 association into the deletion and ts collections in order to identify suppressors. It would have been complex to utilise the GFP-GBP system in the suppressor screens. However to address the reviewers concerns we have made this point clearer by modifying the text:

“Since the forced Cdc5-Mtw1 association during mitosis mildly affected the transition from metaphase to anaphase/telophase despite exhibiting a short spindle phenotype (Fig 3E and S5C Fig), we next asked whether this was the case for the Cdc5-Ame1 association. Also, since both CDC5∆C-AME1 and cdc5∆C-kd-AME1 expression lead to accumulation in metaphase we wanted to assess their ability to transition through mitosis. We induced CDC5∆C-AME1 and cdc5∆C-kd-AME1 expression in metaphase-arrested cells and released them into anaphase using the Cdc20-depletion system as before. Prior to anaphase release, both the control cells containing an empty plasmid and the cdc5∆C-kd-AME1-expressing cells were arrested in metaphase with the Mtw1-YFP forming two separated foci as expected for bioriented spindles, whereas cells expressing CDC5∆C-AME1 often had multiple Mtw1-YFP foci or a scattered signal spread along the mitotic spindle (Fig 4E). Since the kinetochore signal frequently exhibited multiple foci, we deemed it inappropriate to quantify sister-kinetochore distance in these cells as we did for the forced Cdc5-Mtw1 interaction in S5C Fig. This declustered or scattered kinetochore phenotype caused by CDC5∆C-AME1 induction was striking and differed from the cells expressing the cdc5∆C-kd-AME1 fusion, suggesting the phenotype is specific to the kinase activity of Cdc5 and not Ame1 N-terminal fusions. Furthermore, two hours after release from metaphase arrest about 70% of CDC5∆C-AME1 expressing cells remained in metaphase compared to 50% of cdc5∆C-kd-AME1-expressing cells and ~35% of control cells containing an empty plasmid (Fig 4F). The CDC5∆C-AME1-expressing cells also exhibited compromised kinetochores as judged by multiple Mtw1-YFP foci potentially explaining their delayed progression through mitosis (Fig 4F, right panel). However, three hours after metaphase release most of the CDC5∆C-AME1 expressing cells had progressed into anaphase, but with ~30% still remaining in metaphase, whereas both control cells and the cdc5∆C-kd-AME1-expressing cells had continued through mitosis to a greater extent (Fig 4F), consistent with expression of cdc5∆C-kd-AME1 not interfering with anaphase progression.”

In addition, see response to comments 3) and 14); we have now analysed Ame1-GFP cells expressing Cdc5-GBP and show that they accumulate in metaphase to a similar extent as *CDC5∆C-AME1*-expressing cells (S10C Fig).

3) It is unclear whether overexpression of Ame1 can compete out the cdc5∆C-Ame1 and suppress its associated growth defect? It would be useful to know if cdc5∆C kd-Ame1 can complement Ame1 null. Is the essential function of Ame1 intact in this construct? Similarly, I am wondering if Cdc5-Ame1 can complement a cdc5 mutant phenotype when it is not overexpressed.

Ame1 is essential in budding yeast, but we could try to express the kinase-dead *cdc5∆C-kd-AME1* in an *ame1-ts* mutant strain and test whether it can rescue temperature sensitivity, but not in the limited time available for our revision. Regarding the kinase-active *CDC5∆C-AME1* fusion, we would expect it to be lethal even when expressed at intermediate levels. This is partly based on the SPI screening which uses a *CUP1* promoter to express Cdc5-GBP. This promoter induces relatively modest  expression, since we did not add copper in the media. We also used a p*CUP1-CDC5* as a control which is not lethal (in contrast to p*GAL1-CDC5*). Furthermore, we remove the PBD in the direct *CDC5∆C-AME1* fusion and it has been reported that Cdc5 lacking the PBD cannot complement *CDC5* deletion in budding yeast; we would therefore predict that the fusion would not complement a *cdc5* mutant. Regarding the function of Ame1 in the fusion construct we reiterate our response to point 2) above, in particular the *spt4∆* rescue of both Cdc5-GBP in Ame1-GFP (and Okp1-GFP and Mcm21-GFP; all COMA subunits) and the direct *CDC5∆C-AME1* fusion but not other kinetochore associations with Cdc5. In addition, we have now analysed endogenous Ame1-GFP levels in cells expressing either *cdc5∆C-kd-AME1* or *CDC5∆C-AME1* and found that in both cases Ame1-GFP was significantly reduced (S10D Fig), suggesting that endogenous Ame1 is replaced by functional ectopic Ame1 in both fusions at the kinetochore. It has been shown that ectopically expressed tagged kinetochore proteins compete for inclusion into the kinetochore (Herrero and Thorpe. 2016. PLoS Genetics, 12(2): e1005855).  We have adjusted the text within the Results to address points 2) and 3) to make this clearer as follows:

“...since our SPI assays found that a constitutive Cdc5 kinase activity at many different kinetochore subcomplexes produced growth phenotypes (Fig 2E), we asked whether the *spt4∆* suppression was specific to the Cdc5 association with Ame1. For this purpose, we introduced the *pGAL1-CDC5∆C-GBP* plasmid into *spt4∆* strains containing different GFP-tagged kinetochore proteins. To our surprise, none of GFP-tagged kinetochore strains tested (Cse4-GFP internally tagged, Mtw1-GFP, Kre28-GFP, Ndc80-GFP or Dad3-GFP) were suppressed by *spt4∆* (Fig 5D). We were concerned that the *spt4∆* rescue of *CDC5∆C-AME1* growth defect was specific to the genetic fusion construct and not to the forced recruitment of Cdc5 to Ame1. To determine whether this was the case, we introduced the Cdc5∆C-GBP into Ame1-GFP strains with or without *SPT4* . Remarkably, we found that the growth defect was suppressed by *spt4∆* (Fig 5E), and similarly for other COMA complex subunits, Okp1 and Mcm21 (S10A-B Figs). We note that expression of Cdc5∆C-GBP in Ame1-GFP cells accumulated cells in metaphase to a similar extent as *CDC5∆C-AME1*-expressing cells (S10C Fig), and that *AME1* fused to either *CDC5∆C* or *cdc5∆C-kd* can replace endogenous Ame1-GFP at the kinetochore (S10D Fig), consistent with previous data [86], suggesting it is functional. These findings indicate that the phenotype caused by constitutive Cdc5 kinase activity specifically at the COMA complex and not with other kinetochore components requires *SPT4*.”

4) Authors have reported severe kinetochore and spindle defects phenotype that they have contributed to constitutive Cdc5-Ame1 association. However, these phenotypes are hallmarks of defective Dynein function. Ame1 is known to exhibit negative genetic interaction with Dynein (please see, SGD); and fusing Cdc5 with Ame1 may potentially affect its interaction with Dynein components at the kinetochores. In line with this explanation, authors have also observed Dynein components in their list of Cdc5 SPI proteins. It is therefore unclear whether phenotypes observed in this study are contributed solely due to Cdc5-Ame1 fusion or also mediated by defective Dynein function. What are their thoughts on this? Separation of function alleles could be used for future studies.

This is very interesting and we appreciate the comment regarding the Ame1-Dynein negative interaction, which we were not aware of. Interestingly, deletion/inhibition of Dynein/Dynactin components suppressed the *CDC5-AME1* fusion phenotype and also *CDC5* overexpression. We do not see any Dynein components in the list of Cdc5 SPIs (supplemental data 3) as suggested by the reviewer, however, as stated above, dynein components can be found in the list of *CDC5-AME1* and *CDC5* suppressors (supplemental data 4). Notably, in human cells Plk1 is required for Dynein recruitment to kinetochores (Bader et al. 2011. Journal of Biological Chemistry, 286 (23), 20769–20777). This indeed indicates that the Dynein/Dynactin pathway may be involved in the phenotype we observe, which requires further investigation. We have added the following to the discussion to highlight this:

 “We note that deletions and mutations of genes in other complexes also suppressed the growth defect caused by forced Cdc5-Ame1 association, such as Condensin and Dynein/Dynactin components (S9J Fig and S4 Data), suggesting that other pathways are also involved in the phenotype. Interestingly, Ame1 has a negative genetic interaction with Dynein subunits [36]. In human cells, Plk1 is required for Dynein recruitment to kinetochores [106] and a Dynactin subunit can recruit Plk1 to kinetochores [107]. Our data may indicate that Dynein and Dynactin are also involved in Cdc5 function at the budding yeast kinetochore, which requires further investigation.”

5) Authors have proposed a role for Cdc5 in regulation of CEN transcription outside of the S-phase, which includes Cbf1 and/or Spt4. If this conclusion holds true, then conditional depletion of Cdc5 may allow the accumulation of CEN transcripts. It would be interesting to examine the CEN transcript levels outside of the S-phase after depletion of Cdc5 (perhaps GAL-Cdc5).

We failed to make clear that our data suggests that normally Cdc5 elevates transcription during S phase (not outside S phase). However, by conditional recruitment of Cdc5 to the kinetochore, we can indeed drive transcription outside of S phase, causing the problems for the cell. Based on our data, we would predict that Cdc5 depletion would reduce CEN RNA levels, rather than lead to their accumulation; although depletion of Cdc5 will result in pleiotropic effects on cell cycle progression. We did not examine RNA levels at different points in the cell cycle which would have been a nice addition, we have specifically addressed this in the discussion by adjusting the following section:

“Cdc5 is tightly regulated during the cell cycle and its expression begins in S-phase, although its presence at the centromere peaks during mitosis [26]. We did not assess CEN RNA levels at different points in the cell cycle, which will be interesting to explore in future studies. Our data, however, support the notion that  upregulation of CEN transcripts, outside S-phase, causes mitotic defects [89]. Moreover, our data showing an increase in Cse4 signal in response to *CDC5∆C-AME1* induction (S13 Fig) are consistent with Cse4 elevation at centromeres in *cbf1∆* cells [90]. The PBD of Cdc5 has been shown to interact with Cbf1 [25]. Thus, we speculate that Cdc5 may be involved in inhibiting or displacing Cbf1 in a highly regulated manner, during this brief period in S-phase resulting in increased CEN RNA levels. However, since deletion of *CBF1* is viable and the additional Cbf1 recruitment only partially suppressed the *CDC5∆C-AME1* phenotype, we suspect other players are also involved.”

6) Authors have concluded that the growth arrest phenotype caused by forced association of Cdc5 with kinetochore is independent of the SAC. Similar conclusions have been drawn for Cdc5∆C data. However, authors have not examined mad2∆ which has documented evidence of genetic interaction with Cdc5. To better understand the role of SAC, examination of phenotypes in combination with mad2∆ will be helpful. I don’t think that the results will change but I am curious.

This is interesting and retrospectively we should have included a *mad2∆ MTW1-GFP* strain in the experiment shown in Fig S5D.  We tested deletions of *MAD1, MAD3, BUB1* and *BUB3* and deemed it sufficient to assess SAC dependence on the phenotype. We also tested *mad3∆* in multiple kinetochore-GFP strains and found no suppression. We did however test all of the SAC mutants (including *mad2∆*) in the *CDC5-AME1* suppressor screen and none suppressed. We have now added a comment to the discussion that highlights this possibility:

 “Our data suggest that forced Cdc5 recruitment to the kinetochore arrests cell growth in a SAC-independent manner. Polo kinase in many metazoans  is involved in the SAC by facilitating Mps1 activity. We showed that deletion of the SAC component, *MAD3*, did not suppress the growth defect caused by forced Cdc5 recruitment to many different kinetochore components, including the KMN network, which contrasts with Mps1 SPIs [22]. Although we did also test deletions of *MAD1, BUB1* and *BUB3* in cells with forced Cdc5-Mtw1 association we did not test this in a *MAD2* delete. Since *MAD2* has a genetic interaction with *CDC5* [106–108], it would be worth addressing this in future studies. We note that deletions of all SAC components (including *MAD2*) did not suppress the growth phenotype caused by forced association of Cdc5 with Ame1.”

7) For suppressor screen with cdc5∆C-Ame1, the authors used the Cdc5 as a control for lethality. To enrich the suppressors that are specific for Ame1-dependent forced recruitment of Cdc5, I thought it would have been worthwhile to perform the suppressor screen with Cdc5-Ame1 (with intact Polo box) and still use Cdc5 as a control or filter.

The Cdc5∆C-Ame1 suppressor screen mainly served to narrow down genetic dependencies on the growth phenotype. We felt that a positive and a negative control was sufficient to identify Cdc5∆C-Ame1 specific suppressors, and that any additional controls could complicate the results. Polo-kinase fusions are typically used without the PBD, thus we used the same approach. However, we agree that additional controls such as the one the reviewer suggests would be worthwhile to further understand the interaction.

8) Line 18 (abstract): “where”? please correct.

This has been corrected.

9) Line 118: “six additional kinetochore proteins fused to GBP”. Why only these six were selected? Why not other kinetochore proteins from the Table 1? Please provide explanation for this selection.

These six (12 in total) GBP fusions were chosen to represent a single member for each of the kinetochore subcomplexes in the SPI screens. We have used these kinetochore proteins in prior studies, tagged with either fluorescent proteins or GBP with success. The proteins in Table 1 are GFP-tagged kinetochore proteins that were identified in the SPI screen with Cdc5-GBP, which was performed after the kinetochore SPI screens with the 12 kinetochore-GBP fusions. We have adjusted the following sentence to highlight this in the results: “We screened these 12 GBP-fusions, which we chose to represent members of every kinetochore subcomplex, against the collection of 439 GFP strains.”

10) Lines 120-122: Authors tagged proteins with -GBP and -GFP. It is important to determine whether tagging may contribute to the observed SPI. Do tagging of these proteins affect their structure-function, in other words, are there any phenotypes caused by these tags?

As a control in the SPI screens we use GBP alone, which typically does not cause growth defects with GFP-tagged proteins, however there are some GFP strains that were sensitive to GBP alone and those strains were excluded from our analysis. We appreciate this comment and have now made this clearer in the methods section: “We note that some GFP-strains are sensitive to GBP alone which were excluded from the SPI analysis.”

11) Are most of the binary SPI identified with Cdc5 reproducible when reciprocal fusion with GFP and GBP between Cdc5 and targeted kinetochore protein were tested? One would expect that swapping the fusion will more likely not affect the outcome.

This is mostly the case, but we found that ectopic Cdc5-GBP produced more and stronger SPIs with endogenous GFP-tagged kinetochore proteins compared to endogenous Cdc5-GFP with ectopic GBP-tagged kinetochore proteins.This could be partly explained by the ectopic Cdc5 expression. In a normal cell, Cdc5 expression is tightly controlled during the cell cycle. Therefore, the assumption is that premature Cdc5 kinetochore localization is more readily achieved by ectopic Cdc5-GBP (under the control of *CUP1* promoter) compared to Cdc5-GFP driven by the endogenous promoter. This was consistent with our observation using fluorescence microscopy. In addition, any difference can also be explained by the spatial orientation of the GFP-GBP interaction in three dimensions. For example, a kinase domain may not reach a particular substrate in one orientation versus another.

12) Lines 128-129: Why only Cdc5 was selected for further study? It seems like it was based on their previous study with Cdc5, but additional explanations and clarifications would be helpful.

See also the response to the first comment by reviewer #3. We have modified the results section to address this as follows:

 “We chose to further investigate Cdc5 for multiple reasons. First,  we previously identified Cse4 as a substrate of Cdc5 [27]. Second, Cdc5 has been shown to interact with both Cse4 and Ndc80 [25] and phosphorylate microtubule-associated proteins (MAPs) such as Stu2 and Slk19 [40] and the SAC component Mad3 [41]. Third, phosphorylation of Cse4 by both Cdc5 and Ipl1Aurora B kinase is important for faithful chromosome segregation [42]. Fourth, Cdc5 responds to CDK phosphorylation, making it a good candidate for facilitating or amplifying cell cycle signals at the kinetochore. Fifth, phosphorylation of other kinetochore proteins by Cdc5 remains largely unexplored in budding yeast. Finally, in our SPI screen we identified Bbp1 as a SPI with most kinetochore proteins (9/12; S2 Data) and both Nbp1 (a binding partner of Bbp1) and its paralog, Ypr174c, as kinetochore SPIs with a similar SPI phenotype to Cdc5 (Fig 1B) Ypr174c, which is proposed to be an anchoring scaffold for Cdc5 [43], Bbp1 and Nbp1 all interact with Cdc5 [25]. Nbp1 is a component of the Mps2-Bbp1 complex which associates with the SPB and is important for mitotic function of Cdc5 [29]. Interestingly, the S. pombe Bbp1 homolog, Sos7, is a kinetochore component [44]. In S. cerevisiae, Bbp1 interacts with the CCAN proteins, Nkp2 and Mcm22, and Nbp1 and Ypr174c interact with Ame1 and Ctf19, respectively [39,45]. Ame1 and Ctf19 are both components of the COMA kinetochore subcomplex which is part of the CCAN. Our kinetochore SPI data suggest that Cdc5 may function at the inner and the outer kinetochore in agreement with findings in both yeast and human cells, which together suggest it may have multiple functions across the kinetochore [25,30].”

13) Lines 195-196: “ what was striking form these data was the number of kinetochore proteins represented in this set of Cdc5”. Please specify how many kinetochore proteins?

The sentence is now: “What was striking from these data was the number of kinetochore proteins represented in this set of Cdc5 SPIs (18 of the 33 proteins annotated as “kinetochore component”, were in the top 62 SPIs; GO enrichment p value = 3 x 10-15; Table 1).”

14) Lines 300-318: Cell cycle arrest in metaphase observed upon forced Cdc5-Ame1 association could be due to fusion construct, hence this observation is different from previous results for MIND, Cse4 and Dam1/Dash complex. Also, it is unclear why cells accumulate with G1 DNA content despite their metaphase spindle phenotype. Is it possible that cells are stuck in late telophase with aberrant spindles due to issues with cytokinesis. Please explain.

The reviewer is concerned that the N-terminus of Ame1 is perturbed by fusion to Cdc5, resulting in the phenotype (see also the responses to comments 2) and 3)). If this was the case we might expect a similar phenotype for CDC5-AME1 and cdc5-kd-AME1 and indeed expression of both of these result in metaphase delay (Fig 4B), but only the active Cdc5 kinase causes the abnormal kinetochore and spindle morphology  based on microscopy analysis (Fig. 4C and 4D) and perturbs cell growth (Fig 4A). Also, in contrast to cdc5-kd-AME1, flow cytometry showed that CDC5-AME1 expression had a higher frequency of cells with lower DNA content. Collectively, these data show that the fusion of an active Cdc5 kinase to the N-terminus of Ame1 gives a very different phenotype to that of an inactive kinase. Flow cytometry cannot distinguish between G2/M and telophase cells, thus telophase cells would be in the G2/M category. The lower DNA content in cells expressing CDC5-AME1 is intriguing given their metaphase spindle content. We have now tried to make this clearer and highlighted the key difference between spindle morphology and DNA content in the text, as follows:

 “...we also investigated CDC5∆C-AME1 expressing cells by testing the DNA content using flow cytometry. Compared to both an empty plasmid control and cdc5∆C-kd-AME1 expression, cells expressing CDC5∆C-AME1 had less cells with a complete 2C DNA content (S6B Fig). Thus, despite their metaphase spindle appearance, it appears that CDC5∆C-AME1-expressing cells are failing to complete full DNA replication or have an abnormal DNA content. Together these data show that cells expressing CDC5∆C-AME1 have aberrant kinetochore foci consistent with impaired metaphase to anaphase transition and also lower DNA content.”

We also mention this now in the discussion, as follows:

“Additionally, we found that induction of the Cdc5-Ame1 association resulted in a lower DNA content despite cells exhibiting a metaphase appearance (S6B Fig), suggesting that constitutive Cdc5 localization at the COMA complex and/or premature induction of CEN transcription may interfere with DNA replication.”

We have now also analysed Ame1-GFP cells expressing *CDC5∆C-GBP* and they also accumulate in metaphase (see S10C Fig), suggesting that metaphase delay is seen even when the N-terminus of Ame1 is not perturbed.

Also, the rescue of *CDC5-AME1* with *spt4∆* suggests that the N terminus of Ame1 is not specifically perturbed.

15) Line 349: Authors stated that SPI phenotype was minimal for Okp1-GFP and absent for Ctf19-GFP. Curious why Mcm21-GFP was not examined and if it was done, then what phenotype were observed?

This experiment was designed to investigate other kinetochore proteins in *CDC5∆C-AME1* expressing cells. This was not part of the Cdc5 SPI analysis, which did detect Ame1-GFP and Okp1-GFP, but weaker effects with Mcm21-GFP and Ctf19-GFP, depending on which control we use (see S3 Data). For the kinetochore analysis in *CDC5∆C-AME1* expressing cells, we imaged Mtw1, Ndc80, Dad4, Okp1, Ctf19, Mif2 and Cse4 tagged with GFP or YFP. We did not examine Mcm21-GFP. We chose Okp1 which is essential and dimerizes with Ame1 and the non-essential Ctf19 which dimerizes with Mcm21 (also non-essential). They are all members of the COMA complex and we felt that this was sufficient to investigate COMA. We found that CCAN proteins were less affected by *CDC5∆C-AME1* expression than outer kinetochore proteins. However, it is possible that Mcm21 is affected by *CDC5-AME1*, but this would be expected to affect Ctf19-GFP as well since they dimerize, which we did not see in our data.

16) Line 438: Authors have used spt4-138 allele to suppress the Cdc5delC-Ame1 phenotype. What is the status of CEN transcription and whether it is increased in this mutant allele? Also the transcription phenotype was observed for CEN3 and CEN1 but not CEN8. Any explanation for this difference?

We agree that it would be interesting to investigate the *spt4-138* mutant in more detail, especially since it has been shown to cause a more severe chromosome missegregation phenotype than deletion of *SPT4*. However, in the *spt4-138* mutant, a conserved cysteine in the zinc finger domain at position 10 is mutated to tyrosine. Cysteine-10 and other cysteine residues in the zinc finger domain of Spt4 are essential for its function and this mutation possibly inhibits binding to DNA/RNA or Spt5. Therefore, based on studies by others, we would predict that *spt4-138* would behave like the *SPT4* deletion in terms of transcription.

Regarding the differences between *CEN1*, *CEN3* and *CEN8* RNA, we found that expression of *CDC5-AME1* increased *CEN* RNA in all three cases. However, deletion of *SPT4* did not suppress the increased *CEN8* RNA in contrast to *CEN1* and *CEN3*. We do not have a clear explanation for these differences, but it could indicate that centromeres are differentially transcriptionally regulated. This notion has been proposed by Chen et al. 2019. Genetics, 213, 465–479. And notably, *CEN8* and *CEN13* contain an additional Cbf1 binding site. It would be worth exploring these differences in future studies. We have now pointed this out in the text as follows:

”...we found that the increased CEN1 and CEN3 RNA was dependent on *SPT4*, whereas CEN8 was not (Fig 6A). It has been proposed that centromeres are differentially transcriptionally regulated [90], which may explain the difference between CEN1/3 and CEN8. These results indicate that association of Cdc5 to the COMA complex can upregulate CEN RNA levels, which largely depends on the elongation and mRNA-processing factor Spt4.”

17) Figure 1C, the term “Log Growth Ratio” is a little confusing, since, based on the color (yellow to blue), it should be “Log growth inhibition ratio”.

The term Log Growth Ratio (LGR) in this context is used by a number of laboratories to indicate growth effects. Although the term may not be perfect, it has been used in numerous publications and we did not wish to “muddy the waters” in the field. We have added a sentence to clarify that positive LGR indicates a growth defect: “For the kinetochore SPI screens, mean Z-scores or mean log growth ratios (LGRs) were calculated from two comparisons of colony sizes of GFP cells containing GBP-tagged kinetochore protein. The first comparison is with control colonies of the same GFP cells containing GBP alone. The second comparison is with control colonies with the relevant gene not tagged with GBP. These two controls account for the growth defects caused by, first, GBP-GFP binding and, second, ectopic expression of the gene. Consistent, with previous studies, a positive Z-score or LGR indicates a growth defect compared to control colonies and a negative number can either indicate a growth enhancement compared to control colonies or poorer growth on control plates versus experiments. We note that some GFP-strains are sensitive to GBP alone which were excluded from the SPI analysis.”

18) Figure 6A: Although the data for CEN transcription are thoroughly evaluated and appropriately analyzed, it is unclear if this experiment was repeated to confirm the findings.

This experiment was repeated three times but we show one (Fig 6A, left panel) and quantified (right panel) as is typical for this type of experiment. However, we have now included the replicate experiments in the supplementary data (S14A Fig).

19) Grammatical and spelling errors in abstract (lines 13-14 and 18, respectively)

Thank you for pointing out these errors, we have corrected these.

20) Scale bar missing for Figure 3B and 3D.

We have ensured that the scale bars are present in all micrographs.

Reviewer #3: Error-free chromosome segregation relies on the ability of kinetochores to make correct attachments to the spindle microtubules. This fundamentally critical role of kinetochore is tightly controlled by numerous protein-protein interactions often modulated by regulators such as kinases and phosphatases. We have a pretty good understanding of the kinetochore composition and good knowledge on the identities of several kinetochore regulators. However, most kinetochore regulators possess diverse roles involving multiple kinetochore components and as a consequence, dissecting and understanding specific roles of essential regulators often prove difficult. Here, Olafsson and Thorpe employ a Synthetic Protein Interaction (SPI) screen, which was successfully used by the authors in the past to gain insights into the role of Cdc14 and checkpoint activation in budding yeast, to unravel novel roles of Cdc5 (Polo-like kinase) at the kinetochore. They show that forced targeting of Cdc5 to distinct kinetochore subcomplexes resulted in growth phenotype in a kinase dependent manner and the phenotypes vary depending on when and where the Cdc5 was recruited. Particularly, the forced association of Cdc5 with the Constitutive Centromere Associated Network (CCAN) component Ame1 (CENP-U) resulted in kinetochore delustering and impaired metaphase to anaphase transition. Finally, their work also shows that the CCAN recruitment of Cdc5 increased centromeric RNA via Spt4/5 complex, a complex involved transcription elongation and mRNA processing.

In my opinion, this manuscript reports a lot of interesting observations. I do note, that with the employed approach it is hard to confidently rule out possible indirect consequence of constitutive targeting of Cdc5. Having said that, I feel that the authors do a good job of critically analysing their observations in the light of published literature. Overall, I feel that this work will greatly benefit the mitosis community in general and kinetochore field in particular. All my concerns are minor and are outlined below:

• Point 3 line 164 – Not clear how it was decided that cdc5 is important when it is only seen in 3/12 screens using these cut off criteria? Why not cdc28?

See also response to comment 12) from reviewer #2.

Polo-like kinase has multiple roles at the kinetochore in many organisms. In budding yeast, Cdc5 has an elusive kinetochore function which we wanted to explore. Furthermore, the initial screening showed that Cdc5 produced more SPIs with kinetochore proteins (Mtw1, Kre28, Mif2 and Nuf2) than any other kinase, except for Ipl1. Whereas Cdc28 produced two kinetochore SPIs (Dad2 and Mif2). The phenotypic growth effects were also much stronger with Cdc5 compared to Cdc28 (see supplemental data 2).

• Are cdc5 experiments done in haploid and diploid?

Cdc5 experiments were done in haploids only. The initial kinetochore SPI screens were performed in both haploids and diploids.

• Line 356: Might be useful to include a panel with the N-terminal sequence of Ame1 and the Cdc5 consensus sites to appreciate with sites are mutated.

Figure S8 now includes the N-terminal sequence of *AME1* and reported phosphoserines are highlighted. Furthermore, we included the sequences of *AME1* homologs in related yeast species and aligned them to show the conserved regions, some of which include phosphoserines.

• Fig 3, 4E, 4F What size are the scale bars in the yeast images? No scale bars in S10D pictures, but ref in legend.

The size of the scale bars is now included in the legend.

• Figure 4C, please include details of the error bars in figure legend

The details of the error bars are now included in the legend.

• Line 278 – there is no S5E, should be S5D

We have corrected this mistake.

• Fig S5C: how many KTs were quantified? What do the circles represent?

We have clarified the legend to indicate this. The box and whiskers plot indicates the mean sister kinetochore distance and standard deviation of the variance (line and box, respectively). The whiskers indicate the 95 percentile and outliers are indicated as circles. The number of measurements are indicated as n and are shown below the graph.

• Fig 6A (right panel): no error bars?

The graph is a direct measurement of the band intensities in Fig 6A. See response to comment 18) from reviewer #2, we have now included additional examples of these data in S14A.

• Line 529: representative images of Fig 6B would help?

Fig 6B now includes an inset with a representative image.
